# Cytoglobin attenuates pancreatic cancer growth via scavenging reactive oxygen species

**DOI:** 10.1038/s41389-022-00389-4

**Published:** 2022-05-03

**Authors:** Dinh Viet Hoang, Le Thi Thanh Thuy, Hoang Hai, Vu Ngoc Hieu, Kenjiro Kimura, Daisuke Oikawa, Yoshihiro Ikura, Ninh Quoc Dat, Truong Huu Hoang, Misako Sato-Matsubara, Minh Phuong Dong, Ngo Vinh Hanh, Sawako Uchida-Kobayashi, Fuminori Tokunaga, Shoji Kubo, Naoko Ohtani, Katsutoshi Yoshizato, Norifumi Kawada

**Affiliations:** 1 Department of Hepatology, Graduate School of Medicine, Osaka Metropolitan University, Osaka, Japan; 2 Departmet of Anesthesiology, Cho Ray Hospital, Ho Chi Minh City, Vietnam; 3 Department of Hepato-Biliary-Pancreatic Surgery, Graduate School of Medicine, Osaka Metropolitan University, Osaka, Japan; 4 Department of Pathobiochemistry, Graduate School of Medicine, Osaka Metropolitan University, Osaka, Japan; 5 Department of Pathology, Takatsuki General Hospital, Takatsuki, Japan; 6 Department of Pediatrics, Hanoi Medical University, Hanoi, Vietnam; 7 Department of Pain Medicine and Palliative Care, Cancer Institute, 108 Military Central Hospital, Hanoi, Vietnam; 8 Department of Pathophysiology, Graduate School of Medicine, Osaka Metropolitan University, Osaka, Japan; 9 Donated Laboratory for Synthetic Biology, Graduate School of Medicine, Osaka Metropolitan University, Osaka, Japan

**Keywords:** Pancreatic cancer, Oncogenes, Cell growth

## Abstract

Pancreatic cancer is a highly challenging malignancy with extremely poor prognosis. Cytoglobin (CYGB), a hemeprotein involved in liver fibrosis and cancer development, is expressed in pericytes of all organs. Here, we examined the role of CYGB in the development of pancreatic cancer. CYGB expression appeared predominately in the area surrounding adenocarcinoma and negatively correlated with tumor size in patients with pancreatic cancer. Directly injecting 7, 12-dimethylbenz[a]anthracene into the pancreatic tail in wild-type mice resulted in time-dependent induction of severe pancreatitis, fibrosis, and oxidative damage, which was rescued by *Cygb* overexpression in transgenic mice. Pancreatic cancer incidence was 93% in wild-type mice but only 55% in transgenic mice. Enhanced CYGB expression in human pancreatic stellate cells in vitro reduced cellular collagen synthesis, inhibited cell activation, increased expression of antioxidant-related genes, and increased CYGB secretion into the medium. *Cygb*-overexpressing or recombinant human CYGB (rhCYGB) -treated MIA PaCa-2 cancer cells exhibited dose-dependent cell cycle arrest at the G1 phase, diminished cell migration, and reduction in colony formation. RNA sequencing in rhCYGB-treated MIA PaCa-2 cells revealed downregulation of cell cycle and oxidative phosphorylation pathways. An increase in MIA PaCa-2 cell proliferation and reactive oxygen species production by H_2_O_2_ challenge was blocked by rhCYGB treatment or *Cygb* overexpression. PANC-1, OCUP-A2, and BxPC-3 cancer cells showed similar responses to rhCYGB. Known antioxidants N-acetyl cysteine and glutathione also inhibited cancer cell growth. These results demonstrate that CYGB suppresses pancreatic stellate cell activation, pancreatic fibrosis, and tumor growth, suggesting its potential therapeutic application against pancreatic cancer.

## Introduction

Patients with pancreatic cancer (PC) have a 5-year overall survival rate of only 7% due to its early metastasis, insensitivity to chemotherapy, and high recurrence [1–4]. Of the different types of PC, pancreatic ductal adenocarcinoma (PDAC) is the most common and is also one of the most highly malignant cancers.

PC progression is a complex and dynamic process involving interactions between cancer cells and stroma [5], which contains heterogeneous stromal cell populations including pancreatic stellate cells (PSCs), fibroblasts, macrophages, lymphocytes, and endothelial cells [6]. First described ~40 years ago [7], PSCs are considered to be critical in driving PC biology. In pancreatitis, pancreatic fibrosis, and PC, quiescent PSCs storing vitamin A transform into an activated alpha smooth muscle actin (αSMA)-positive phenotype with enhanced contractile activity, migratory capacity, and extracellular matrix (ECM) synthesis as well as the acquisition of an expansive secretome [8]. Activated PSCs induce a strong desmoplastic reaction or stromal fibrosis that is similar to or even stronger than the fibrotic reaction in liver cancers [9–11]. Although PSCs are assumed to regulate the malignancy potential of PC cells, the underlying mechanism remains elusive [12–14].

The production of reactive oxygen species (ROS), including hydroxyl ion (HO^−^), superoxide (O_2_^−^), and hydrogen peroxide (H_2_O_2_), is increased by genetic alterations [15] and metabolic changes [16] during pancreatitis and fibrosis, which can lead to the development of PC [17]. ROS not only induce genomic instability [18] and enhance tumor cell proliferation [19–21] but also may be causative of pancreatic fibrosis [22] and PSC activation [23, 24], which may further promote the growth, survival, and invasion of cancer cells [25, 26]. ROS trigger the activation of PSCs and lead to augmented synthesis of ECM via activation of mitogen-activated protein kinases [27] and secretion of soluble factors such as interleukin-6, stromal cell-derived factor 1, and vascular endothelial growth factor A to favor the invasion of PC [28].

In this regard, antioxidants have been employed to investigate the role of ROS in PC development and PSC activation. For instance, curcumin inhibits the proliferation of MIA PaCa-2 PC cells in vitro and decreases MIA PaCa-2 orthotropic tumor growth in mice by attenuating the redox-responsive transcription factor nuclear factor kappa-light-chain-enhancer of activated B cells [29]. Also, vitamin E suppresses PANC-1, MIA PaCa-2, and BxPC3 PC cell growth in vitro [30, 31] and blocks PC progression in mice expressing oncogenic Kras^G12D^ [32].

Cytoglobin (CYGB) is a 21-kDa protein that shares ~25% identity with vertebrate myoglobin and hemoglobin and 16% identity with human neuroglobin [33]. CYGB is expressed in pericytes of all organs [34], such as hepatic stellate cells (HSCs) in the liver, renal cortical interstitial fibroblast-like cells, stromal cells of red pulps in the spleen, and PSCs in the pancreas, but not in epithelial cells, endothelial cells, muscle cells, blood cells, macrophages, or dermal fibroblasts. We previously demonstrated that the loss of CYGB promotes tumors in multiple organs in aged mice [35] and that CYGB plays a key regulatory role in HSC activation via ROS scavenging.

In the present study, we investigated the role of CYGB in PC development and PSC activation. We show (i) a negative correlation between CYGB expression and tumor stage in human PC tissue; (ii) suppression of 7,12-dimethylbenz[a]anthracene (DMBA)-induced PC in *mCherry* reporter-specific *Cygb*-overexpressing transgenic (TG) mice compared with wild-type (WT) mice; (iii) attenuation of PSC activation and collagen production in human PSCs overexpressing *CYGB*; and (iv) reduced human PC cell proliferation, colony formation, and cell migration under in vitro *CYGB* overexpression or recombinant human (rh)CYGB treatment; (v) the mechanism of cancer cell growth inhibition via cell cycle arrest under the ROS scavenging function of CYGB.

## Results

### Dominant CYGB expression in the area surrounding PDAC and its negative correlation with tumor stage

**Fig. 1:**
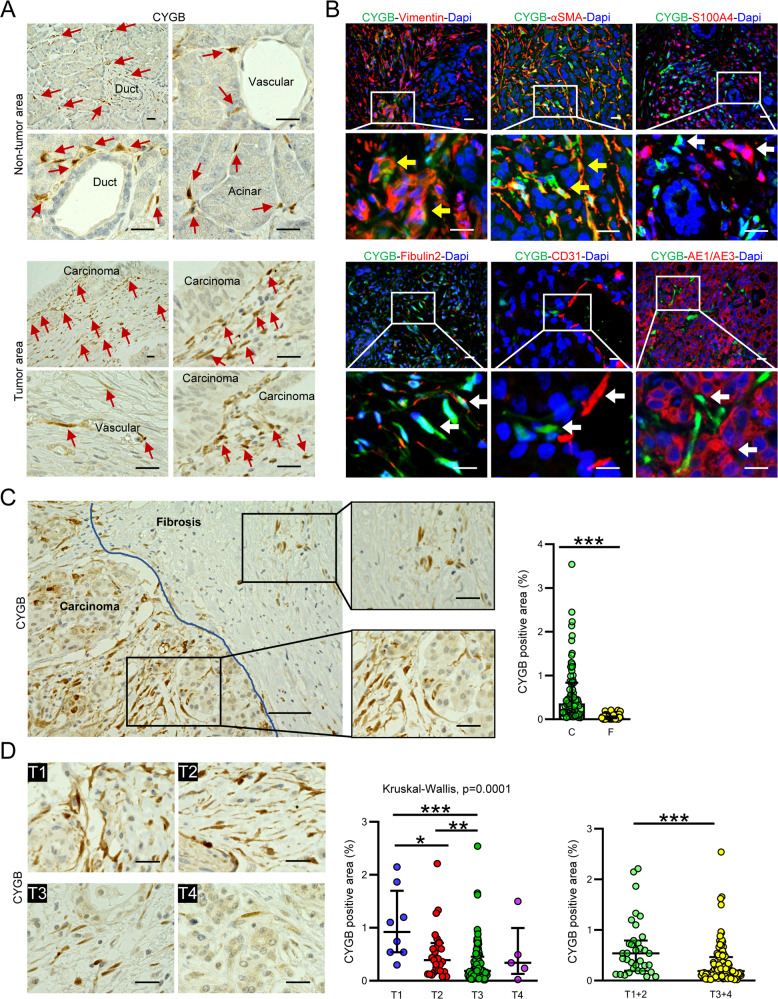
CYGB expression in pancreatic ductal adenocarcinoma is negatively correlated with tumor stages. **A** Representative immunohistochemical staining images of human pancreatic cancer tissues for Cytoglobin (CYGB) in non-tumor and tumor area. Arrows, CYGB positive cells. Scale bars, 20 µm. **B** Double immunofluorescent detection of CYGB (green) with other cells’ makers (red), including pancreatic stellate cells (αSMA, Vimentin), fibroblasts (S100A4, Fibulin 2), endothelial cells (CD31), and epithelial cells (AE1/AE3). Yellow arrows indicated CYGB co-localization with other markers; white arrows indicated single positive cells. Scale bars, 20 µm. **C** Representative immunohistochemical staining for CYGB and their quantifications (right panel) in fibrosis and carcinoma area of hPDAC, *n* = 86 pairs. Scale bars, 50 µm. Scale bars in magnificent image, 20 µm. **D** Immunohistochemical detection of CYGB and the quantification of its expression in hPDAC with T stages from 1–4. Right insets, quantification charts. *n* = 157. Scale bars, 20 µm. Hematoxylin (**A**, **C**, **D** panels) and 4′,6-diamidino-2-phenylindole (Dapi, **B**) were used to visualize nuclei. Data were shown as medians and interquartile ranges. *p < 0.05, **p < 0.01, ***p < 0.001, Mann–Whitney *U* tests, two-tailed.

As CYGB was originally identified in rat HSCs [36], we recently determined its expression and role in liver fibrosis and cancer [37, 38]. However, the role of CYGB in human PC remains undetermined. We first assessed CYGB expression in non-tumor and tumor areas of human (h)PDAC tissue exhibiting malignant ductal proliferation surrounded by dense ECM, αSMA-positive myofibroblasts, and cancer cells positive for the DNA damage markers P53 and 53BP1 (Supplemental Fig. 1A). In the non-tumor area, CYGB positivity localized in peri-ductal, peri-vascular, and peri-acinar cells (Fig. 1A, top panels). In the tumor area, CYGB-positive cells were mostly found surrounding adenocarcinoma (Fig. 1A, bottom panels). Immunofluorescence analysis revealed that CYGB positivity co-localized with vimentin and αSMA (Fig. 1B), which are well-known markers of PSCs [39, 40], but negligibly co-localized with S100A4 and fibulin 2, which are markers of myofibroblasts other than PSCs [41]. CYGB was not expressed in CD31-positive endothelial cells or AE1/AE3-positive pancreatic carcinoma (Fig. 1B). These results clarify that CYGB-positive cells in hPDAC tissue are PSCs.

We further assessed CYGB expression in hPDAC tissue from 157 patients, including 8, 24, 120, and 5 patients classified as Stage T1, T2, T3, and T4, respectively, as assessed using the tumor-node-metastasis pancreatic tumor classification system described in the 8^th^ edition of the Union for International Cancer Control staging system (Supplemental Table 1). CYGB-positive cells were more abundant in carcinoma areas than in ECM-rich fibrotic septum in all samples (Fig. 1C). When quantified based on total sample area, we did not find a correlation between CYGB expression and patient survival. We used Spearman’s rank correlation test to determine correlations between the degree of CYGB positivity and overall survival (OS) and applied Kaplan–Meier analysis to compare OS between groups (Supplemental Fig. 1B). Receiver operating characteristic curve analysis was used to determine the most appropriate cutoff values. CYGB positivity detected in ≤ 0.154% of the tissue section was defined as low CYGB expression. Although no correlations were identified between CYGB expression levels and survival time, the highest CYGB positivity was found at T1 stage, and the lowest positivity was found at T3 and T4 stages (Fig. 1D, left and middle panels). When combined into two groups, CYGB positivity was lower in the T3 + 4 group than in the T1 + 2 group (Fig. 1D, right panel). These results show that CYGB was expressed in PSCs in both non-tumor and tumor areas of hPDAC tissue but was nearly absent in fibrotic septum, and its positivity was negatively correlated with tumor stage.

### Attenuation of DMBA-induced pancreatic tumorigenesis by *Cygb* overexpression in mice

**Fig. 2:**
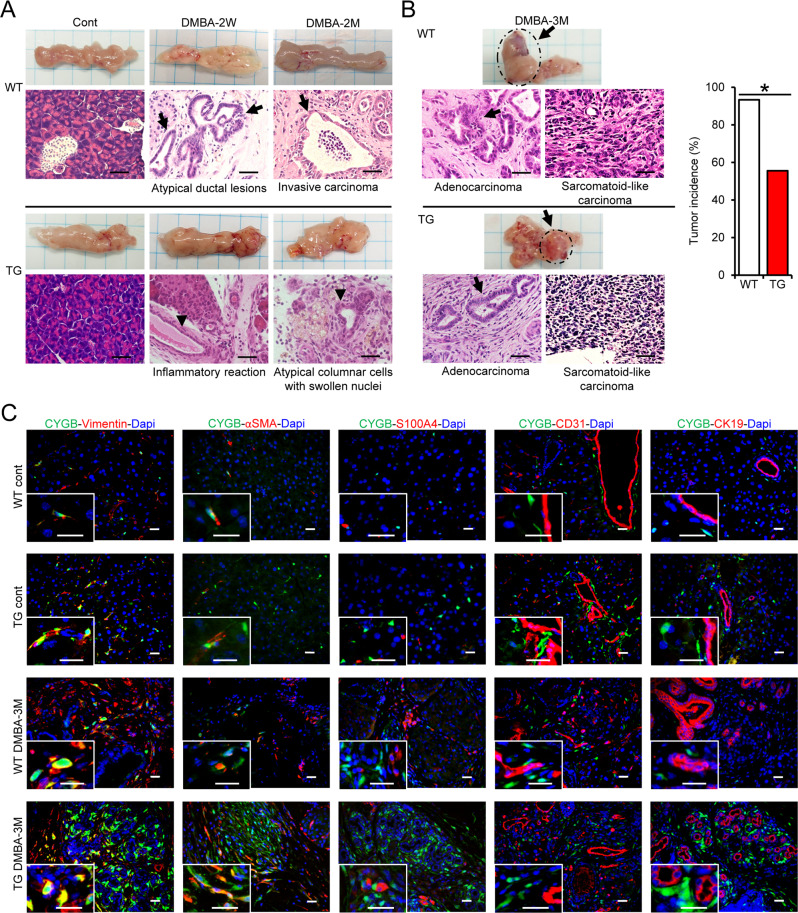
Overexpression of *Cygb* attenuated DMBA-induced pancreatic tumorigenesis in mice. Wild-type (WT) and *Cygb* with *mCherry* reporter-overexpressing (TG) mice were under placebo (cont) or DMBA treatment at different time-points. **A** Representative pancreatic macroscopic and microscopic H&E staining images. *N* = 3 in control groups and *n* = 8 in DMBA treatment groups. Scale bars, 20 µm. **B** Representative pancreatic macroscopic and microscopic H&E staining images in 3-month DMBA-treated mice and their tumor incidence. *N* = 15 in WT group and *n* = 9 in TG groups Scale bars, 20 µm. *p < 0.05, Fisher’s exact test, two-tailed. **C** Double immunofluorescent staining for CYGB (green) and markers for PSCs (Vimentin), activated PSCs (αSMA), fibroblasts (S100A4), endothelial cells (CD31), and pancreatic ductal cells (CK19) in mouse pancreatic tissues. Dapi was used to visualize nuclei. Scale bars and inset, 20 µm.

To test the hypothesis that CYGB negatively regulates PC progression, we challenged TG and WT mice with 1.5 mg DMBA injected directly into the pancreatic tail. The pancreas of WT mice showed ductal proliferation with hyperchromatic nuclei and irregular shape 2 weeks after DMBA injection, which was followed by the development of invasive carcinoma (Fig. 2A, arrows) associated with a desmoplastic reaction at 2 months (Fig. 2A, top panels). At 3 months, pancreatic tumors were macroscopically observed and microscopically composed of both ductal adenocarcinoma and sarcomatoid-like carcinoma (Fig. 2B). By contrast, TG mice showed only an inflammatory reaction 2 weeks after DMBA injection and the proliferation of atypical columnar ductal cells with swollen nuclei at 2 months (Fig. 2A, bottom panels, arrowheads). Tumor incidence was lower in TG mice than in WT mice; 5 out of 9 (55%) TG mice and 14 out of 15 (93%) WT mice had tumors at 3 months (Fig. 2B, right panel).

Tumor volume tended to be lower in DMBA-treated TG mice compared with DMBA-treated WT mice, but this difference was not statistically significant (Supplementary Fig. 2A). An adenocarcinoma pattern was observed in 80% (12 out of 15) of WT mice and 22% (2 out of 9) of TG mice, whereas a mixture of adenocarcinoma and sarcomatoid-like carcinoma was observed in 13% (2 out of 15) of WT mice and 33% (3 out of 9) of TG mice (Supplemental Fig. 2B). Immunohistochemical staining showed the presence of cells positive for Ki67, a proliferation marker, in both adenocarcinoma and sarcomatoid-like carcinoma areas in DMBA-treated TG and WT mice at 3 months (Supplemental Fig. 2C).

CYGB expression was concomitant with mCherry expression in the PSCs of TG mice (Supplemental Fig. 2D). CYGB expression in mouse PSCs was confirmed by double immunofluorescence staining of vimentin and αSMA (Fig. 2C). Similar to our observations in hPDAC tissue, CYGB-positive cells negligibly co-localized with S100A4, a marker of fibroblasts; CD31, a marker of endothelial cells; or CK19, a marker of pancreatic ductal cells.

### Attenuation of DMBA-induced pancreatic inflammation and fibrosis by *Cygb* overexpression in mice

**Fig. 3:**
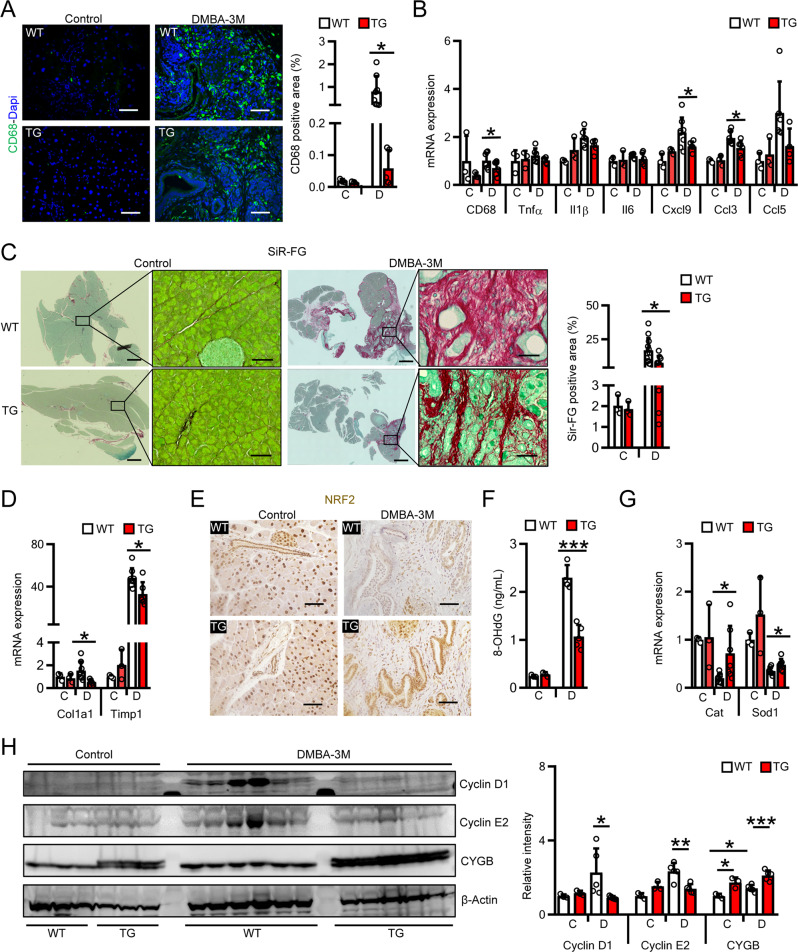
Overexpression of *Cygb* attenuated DMBA-induced pancreatic inflammation and fibrosis in mice. **A**, **B** Representative immunofluorescent staining images of pancreatic tissues for macrophages (CD68) in mice with DMBA-induced PC or placebo at 3-month time point along with their quantifications (**A**), and their qRT-PCR analysis of inflammation related-genes (**B**). Scale bars, 20 µm. **C** control; **D** DMBA-3M. Dapi was used to visualize nuclei. **C** Representative Sirius red and fast green (SiR-FG) staining images of pancreatic tissues in mice with DMBA-induced PC or placebo at 3-month time point along with their quantifications. Scale bars, 500 µm; inset scale bars, 20 µm. **C** control; **D** DMBA-3M. **D** qRT-PCR analysis of fibrosis related-genes in mice with DMBA-induced PC at 3-month time point. **C** control; **D** DMBA-3M. **E** Representative images of immunohistochemical staining for NRF2 in pancreatic tissues of mice with DMBA-induced PC or placebo at 3-month time point. Hematoxylin was used to visualize nuclei. **F** 8-OHdG levels in DNA isolated from pancreatic tissues of DMBA or placebo-treated at 3-month time point. **C** control; **D** DMBA-3M. **G** qRT-PCR analysis of antioxidant related-genes in mice with DMBA-induced PC or placebo at 3-month time point. **C** control; **D** DMBA-3M. **H** Immunoblotting analysis and their quantifications of proteins related to cell cycle in mice with DMBA-induced PC or placebo at 3-month time point. β-Actin was used as a loading control. **C** control; **D** DMBA-3M. Data were shown as mean ± SD. *p < 0.05, **p < 0.01, ***p < 0.001, Student’s *t* test, two-tailed.

As inflammatory cells promote PC cell growth and invasion [42], we investigated the infiltration of macrophages in our TG mouse model. The number of CD68-positive macrophages in pancreatic tissue was lower in DMBA-treated TG mice than in DMBA-treated WT mice at 3 months (Fig. 3A). Compared with DMBA-treated WT mice, DMBA-treated TG mice showed lower mRNA levels of CD68, a marker of macrophages; Cxcl9, a four-chemokine signature in primary and metastatic PC; [43] and Ccl3, which plays an important role during chronic pancreatitis [44] (Fig. 3B). No significant changes were observed in mRNA levels of Tnfα, Il1β, Il6, or Ccl5.

There are at least two types of cancer-associated fibroblasts (CAFs) in PC: myofibroblasts (myCAFs) and inflammatory fibroblasts (iCAFs) [45]. We found that CYGB was expressed in myCAFs (Fig. 2C, CYGB^+^Desmin^+^ or CYGB^+^αSMA^+^). However, CYGB-positive cells did not co-localize with iCAFs positive for CD68 (Supplemental Fig. 2E) or interleukin-6 (Supplemental Fig. 2F). Thus, the suppression of an inflammatory reaction in TG mice may reflect the outcome of deactivated PSCs (i.e., myCAFs) under CYGB overexpression, leading to a reduction in cytokine/chemokine-attracting macrophages.

Similar to the predominant fibrosis observed in hPDAC tissue (Supplemental Fig. 1A), marked pancreatic ECM deposition was observed in DMBA-treated WT mice compared with untreated WT mice at 3 months, was reduced by 50% in DMBA-treated TG mice (Fig. 3C). Consistently, downregulation of collagen 1a1 (−63%) and Timp1 (−31.9%) mRNA was found in the pancreas of DMBA-treated TG mice as compared with DMBA-treated WT mice (Fig. 3D).

DMBA induces oxidative DNA damage [46, 47] and CYGB has a ROS scavenging function [36, 48]. Therefore, we assessed the pancreatic expression of nuclear factor erythroid 2–related factor 2 (NRF2), which regulates cellular resistance to oxidants, using immunohistochemical staining (Fig. 3E). Higher NRF2 staining was observed in TG mice than in WT mice following DMBA treatment. We next measured levels of pancreatic 8-OHdG, an ROS-induced DNA damage marker, using ELISA with isolated DNA. DMBA induced a 10-fold increase in 8-OHdG levels in WT mice (2.29 ng/mL) compared with untreated WT mice (0.24 ng/mL), but DMBA-treated TG mice showed lower 8-OHdG levels (−53%) than DMBA-treated WT mice (Fig. 3F). In addition, DMBA-treated TG mice showed higher expression of the antioxidant genes catalase (Cat; 3.5-fold increase) and superoxide dismutase 1 (Sod-1; 1.36-fold increase) than DMBA-treated WT mice (Fig. 3G).

We further investigated the RAS/MARK pathway, by assessing phosphorylated and total ERK expression after DMBA treatment. Immunoblotting analysis showed that ERK phosphorylation was reduced in DMBA-treated TG mice compared with DMBA-treated WT mice at 2 weeks (Supplemental Fig. 3). Consistently, cyclin D1 and cyclin E2 expression were increased in DMBA-treated WT mice, but these increases were rescued in DMBA-treated TG mice (Fig. 3H). Taken together, these results indicate that *Cygb* overexpression may attenuate DMBA-induced tumorigenesis via the suppression of inflammatory and fibrotic reactions, oxidative DNA damage, and oncogene expression in mice.

### Inhibition of PSC activation and collagen production by *Cygb* overexpression in vitro

Our observations thus far suggest that *Cygb* overexpression serves a protective function in chronically damaged pancreatic tissue. Because CYGB is exclusively expressed in PSCs in the pancreas, we hypothesized that CYGB plays a key role in the activation of PSCs, thereby controlling tissue inflammation, fibrosis, and oxidative stress. Therefore, we used a human PSC cell line, HPaSteCs, to test this hypothesis.

HPaSteCs were cultured in medium containing 1% stellate cell growth supplement (S+; ScienCell Research Laboratories). We previously found that a human HSC cell line, HHSteCs, cultured in S+ medium showed increased CYGB protein levels and decreased αSMA protein levels [49]. Whereas HHSteCs appeared flattened and polygonal in shape with thick bundles of stress fibers in S- medium, they exhibited clear boundaries with thinner cell bodies and dissolved stress fibers in S+ medium [49].

**Fig. 4:**
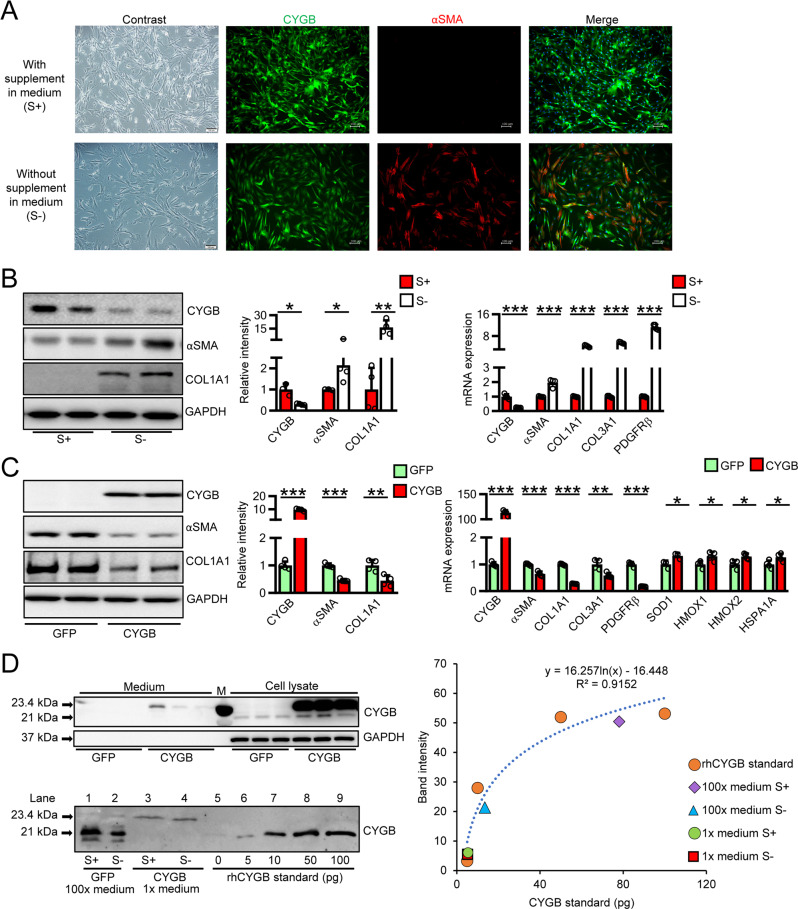
CYGB regulates HPaSteCs activation and collagen production. **A** Representative contrast and fluorescent images of the double staining for CYGB (green) and αSMA (red) in HPaSteCs cultured in medium with (S+) or without (S−) supplement. Scale bars, 100 µm. Dapi was used to visualize nuclei. **B** Immunoblotting analysis of CYGB, COL1A1, and αSMA along with their quantifications (left and middle panel), and qRT-PCR analysis of CYGB, αSMA, COL1A1, COL3A1, and PDFGRβ (right panel) in HPaSteCs under S+ or S− condition. GAPDH was used as loading control. **C** Immunoblotting analysis of CYGB, COL1A1, and αSMA along with their quantifications (left and middle panel), and qRT-PCR analysis of CYGB, αSMA, COL1A1, COL3A1, PDFGRβ, and antioxidant related-genes (SOD1, HMOX1/2, HSP1A1) of HPaSteCs transfected with control lentiviral eGFP expression vector (GFP) or CYGB overexpression vector (CYGB) (right panel). GAPDH was used as loading control. **D** (Top-left panel) Determination of CYGB presence in cell lysate and cultured medium of HPaSteCs by immunoblotting assay. Entire medium (7.5 µL/lane) and total cell lysate (6 µg/lane) from HPaSteCs transfected with control lentiviral eGFP expression vector (GFP) or CYGB expression vector (CYGB) were loaded per lane. His-FLAG-tagged-CYGB (23.4 kDa) presented above endogenous CYGB (21 kDa) in the cell lysate. GAPDH was used as loading control. M molecular marker. (Bottom-left panel) Quantification for CYGB expression by immunoblotting assay. Loading amount: 7.5 µL of 100 time-condensed (100x) (lane 1, 2) or 1x medium (lane 3, 4) from lentiviral eGFP expression vector (lane 1, 2) or CYGB expression vector-transfected HPaSteCs (lane 3, 4) at S+ and S− conditions, along with a ladder of rhCYGB serving as standard samples (lane 5–9). Note that the secreted CYGB from HPaSteCs transfected with control vector (GFP) at S+ and S− conditions (lane 1, 2) were only found when the medium was 100x. (Right panel) A calibration curve was plotted from densities of rhCYGB standards (orange circles) and used to determine the endogenous CYGB content in the 100x medium from HPaSteCs transfected with Lentiviral eGFP vector at S+ (purple rhombus) and S− conditions (blue triangle), and secreted CYGB from entire medium of HPaSteCs transfected with lentiviral CYGB expression vector at S+ (green circle) and S− conditions (red square) (right-panel). Data were shown as mean ± SD from three independent experiments, *n* = 4. *p < 0.05, **p < 0.01, ***p < 0.001, Student’s *t* test, two-tailed.

In the present study, we found that CYGB was expressed in HPaSteCs at the RNA and protein level when cultured in S+ medium (Fig. 4A, B). In sharp contrast, in S− solution, CYGB was downregulated; αSMA, COL1A1, COL3A1, and PDGFRβ were upregulated at the mRNA level; and αSMA and COL1A1 were upregulated at the protein level (Fig. 4B). Thus, similar to our finding in HHSteCs [49], S+ medium regulated the activation status of HPaSteCs.

We previously found that fibroblast growth factor 2 (FGF2) included in S+ medium is a key factor controlling the phenotype of HHSteCs [49]. Similarly, the addition of FGF2 at 4 ng/mL for 48 h induced CYGB expression and reduced αSMA and COL1A1 expression in HPaSteCs (Supplemental Fig. 4). Furthermore, stable overexpression of *Cygb* via lentiviral vector in HPaSteCs exerted the same effect as FGF2-containing S+ medium, resulting in the upregulation of antioxidant genes including SOD1, heme oxygenase 1/2 (HMOX-1/2), and heat shock protein family A (Hsp70) member 1A (HSPA1A) compared with lentiviral eGFP-expressing vector (Fig. 4C). Taken together, these results suggest that CYGB plays an important role in modulating the activation and redox status of HPaSteCs.

### Secretion of CYGB from endogenously CYGB-overexpressing PSCs

Previous in vitro and in vivo studies provide strong evidence of an interaction between PSCs and PC cells in the cancer microenvironment [50–53]. In the present study, we investigated the role of CYGB in PSCs in PC development because we observed a lower incidence of tumors in DMBA-treated TG mice and hypothesized that factors secreted from CYGB-positive PSCs disturb PC development. We examined the medium of HPaSteCs that stably overexpressed *Cygb* via lentiviral vector and found that it contained secreted CYGB protein (bands indicating His-Flag-tagged-CYGB at 23.4 kDa), which was absent from the medium of HPaSteCs transfected with lentiviral eGFP vector (Fig. 4D, top left panel). Based on the calibration curve plotted from densities of rhCYGB standards (Fig. 4D, right panel), we detected CYGB protein levels of 0.052 and 0.009 pg/µL in HPaSteC S+ and S− mediums, respectively (Fig. 4D, bottom left panel, lanes 1 and 2). When CYGB was overexpressed via lentiviral vector, secreted CYGB increased to 0.34 and 0.36 pg/µL in S+ and S− mediums, respectively (Fig. 4D, bottom left panel, lanes 3 and 4). These results suggest that CYGB is secreted from HPaSteCs that endogenously over-express CYGB.

### Effect of exogenous rhCYGB treatment on MIA PaCa-2 cell function

**Fig. 5:**
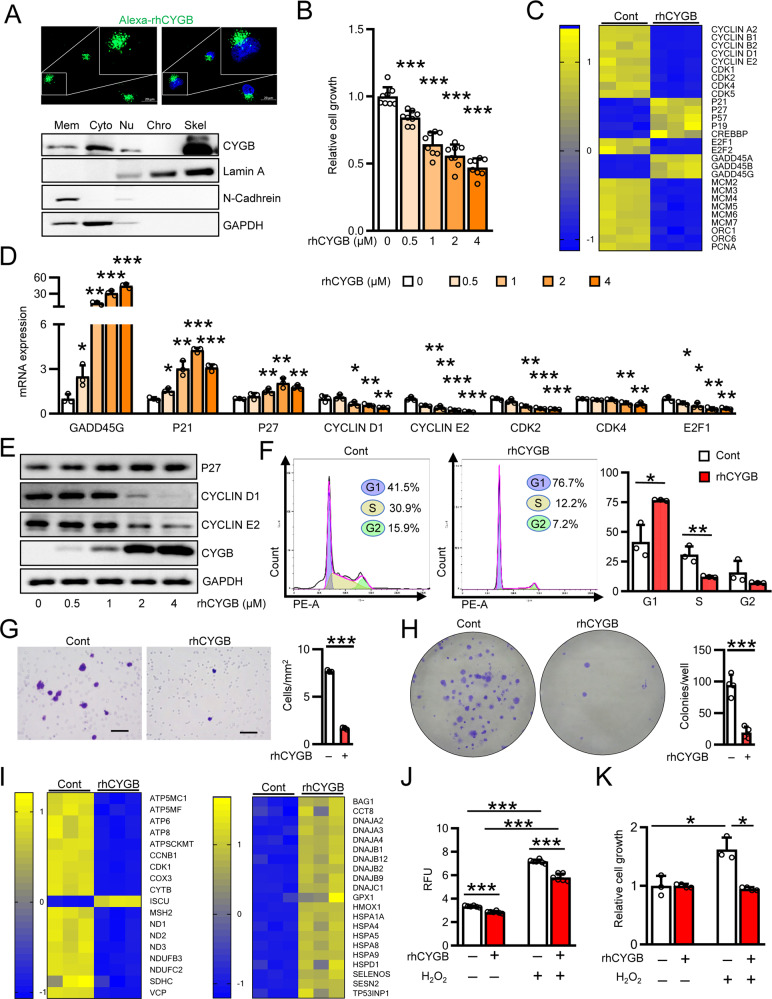
rhCYGB suppressed proliferation, migration, and colony formation of human pancreatic cancer cells. **A** (Top panel) Representative fluorescent images of the intracellular distribution of Alexa 488–rhCYGB (green, 10 µg/mL, 24 h) in MIA PaCa-2 cells. Hoechst 33258 (blue) was used to visualize nuclei. Scale bars, 20 µm. (Bottom panel) Immunoblotting analysis for fractionated cellular proteins. Marker for cytoplasmic (Cyto): GAPDH, plasma membrane (Mem): N-Cadherin, nuclear soluble (Nu) and chromatin-bound (Chro) and cytoskeletal (Skel): Lamin A. **B** Determination of cell proliferation by CCK-8 assay in MIA PaCa-2 cells under rhCYGB-treatment in a dose-dependent manner for 48 h, *n* = 8. **C**, **D** Heatmap analysis of significantly changed cell cycle-related genes in MIA PaCa-2 treated with rhCYGB (4 µM, 48 h) compared to untreated controls by RNA-Seq (*n* = 3) (**C**), and their qRT-PCR analysis (**D**). **E** Immunoblotting analysis of cell cycle-related proteins (P27 and Cyclin D1 and Cyclin E2) in MIA PaCa-2 treated with rhCYGB in a dose-dependent manner for 48 h. GAPDH was used as loading control. **F** Flow cytometric determination of cell cycle distribution in rhCYGB-treated MIA PaCa-2 (2.5 µM, 48 h) and controls, *n* = 3. **G**, **H** Determination of cell migration for 24 h (**G**) and colony formation for 14 days (**H**) in rhCYGB-treated MIA PaCa-2 (2.5 µM) and controls, *n* = 4. Scale bars, 50 µm. **I** Heatmap analysis of oxidative phosphorylation related-genes (left panel) and antioxidant related-genes (right panel) that were changed significantly in MIA PaCa-2 treated with rhCYGB (4 µM, 48 h) compared to untreated controls by RNA-Seq (*n* = 3). **J** Determination of ROS level by DCFDA assay in MIA PaCa-2 treated with or without rhCYGB 2.5 µM for 24 h concomitant H_2_O_2_ 500 µM administration in 1 h, *n* = 6. **K** Determination of cell proliferation by CCK-8 assay in MIA PaCa-2 treated with or without rhCYGB 2.5 µM concomitant with H_2_O_2_ 40 µM for 4 h, *n* = 4. Data were shown as mean ± SD from three independent experiments. *p < 0.05, **p < 0.01, ***p < 0.001, Student’s *t* test, two-tailed. Cont untreated control, RFU relative fluorescent units.

As we found that CYGB was secreted into the extracellular space from PSCs, we next sought to verify the effects of exogenous CYGB on PC cell function. We generated rhCYGB [48], labeled it with Alexa-488, and traced its intracellular biodistribution in MIA PaCa-2 cells for 24 h (Fig. 5A, top panels). Alexa-488-labeled rhCYGB showed clear cytoplasmic localization, similar to the pattern observed in HHSteCs, suggesting that rhCYGB was internalized by cells via the clathrin-mediated endocytosis pathway [48]. We further performed cellular fractionation and found that rhCYGB was present in the membrane, cytoplasm, nuclear, and skeleton fractions (Fig. 5A, bottom panels). MIA PaCa-2 cell proliferation was dose-dependently suppressed by rhCYGB, with an IC_50_ value of 2.5 µM (Fig. 5B).

To understand the underlying mechanism, we performed RNA-seq analysis of untreated and rhCYGB (4 μM)-treated MIA PaCa-2 cells (*n* = 3 per group) and found that the expression of 3721 genes was changed by 2-fold or higher in the rhCYGB-treated group (Supplemental Fig. 5A). Gene set enrichment analysis showed that gene ontology terms associated with biological processes, such as “nuclear division”, “positive regulation of cell cycle”, and “DNA replication”, were overrepresented among differentially expressed genes (Supplemental Fig. 5B).

rhCYGB treatment downregulated cyclins and cyclin-dependent kinases (CDK1, 2, 4, and 5), transcriptional factor E2F1/2, and minichromosome maintenance complex component (MCM) 2–7, which stimulates cell cycle progression, and upregulated cyclin-dependent kinase inhibitor (CDKN) factors such as CDKN1A (P21), CDKN1B (P27), CDKN1C (P57), CDKN2D (P19), and growth arrest and DNA damage inducible 45 alpha/beta/gamma (GADD45A/B/G) (Fig. 5C). qRT-PCR confirmed the dose-dependent downregulation of cyclin D1, cyclin E2, CDK2/4, and E2F1 by up to 80% and upregulation of P21, P27, and GADD45G by up to 50-fold (Fig. 5D). At the protein level, rhCYGB treatment boosted the expression of P27 and suppressed the expression of cyclin D1 and cyclin E2 in a dose-dependent manner in MIA PaCa-2 cells (Fig. 5E). Similar rhCYGB-induced inhibition of cell proliferation was found in other PC cell lines including PANC-1 and OCUP-A2 cells (Supplemental Fig. 6). In addition, two well-known antioxidants, N-acetyl-L-cysteine and glutathione, displayed a similar capacity to suppress MiaPaCa-2 cell growth and the expression of cell cycle–related genes (Supplemental Fig. 7A–D). Combination treatment of rhCYGB for 48 h and NAC for the last 24 h suppressed MIA PaCa-2 cell growth in an additive manner compared to single NAC administration (Supplementary Fig. 7E–F). These observations indicate that the effect of rhCYGB is due to its antioxidant property and ROS is involved in the growth of MIA PaCa-2 cells.

When we assessed the cell cycle phases of MIA PaCa-2 cells using flow cytometry, we found that 76.7% of rhCYGB-treated but only 41.5% of untreated cells were arrested at the G1 phase (Fig. 5F). Moreover, the number of migrated MIA PaCa-2 cells was reduced by nearly 5-fold after 24 h of rhCYGB treatment (Fig. 5G). Colony formation assay also revealed a reduction in the number of colonies following rhCYGB (2.5 µM) addition to fresh medium every 3 days (Fig. 5H).

RNA-seq analysis further revealed that rhCYGB administration (4 µM) for 48 h downregulated the expression of genes involved in the oxidative phosphorylation pathway in MIA PaCa-2 cells (Fig. 5I, left heatmap) such as ATP synthase membrane subunit c locus 1 (ATP5MC1), cytochrome c oxidase subunit III (COX3), and NADH dehydrogenase subunit (ND) 1/2/3, whereas it upregulated the expression of antioxidant genes (Fig. 5I, right heatmap) such as glutathione peroxidase (GPX) 1 and heme oxygenase 1 (HMOX1). The addition of rhCYGB (2.5 μM) reduced ROS production spontaneously and under stimulation by 500 µM H_2_O_2_ for 4 h (Fig. 5J) or 200 µM H_2_O_2_ for 1 h (Supplemental Fig. 8A) and suppressed cell proliferation under stimulation by 40 µM H_2_O_2_ (Fig. 5K). These results suggest that extracellular CYGB treatment scavenged ROS and prohibited PC cell growth and migration in vitro.

### Effect of endogenous Cygb overexpression on MIA PaCa-2 cell function

**Fig. 6:**
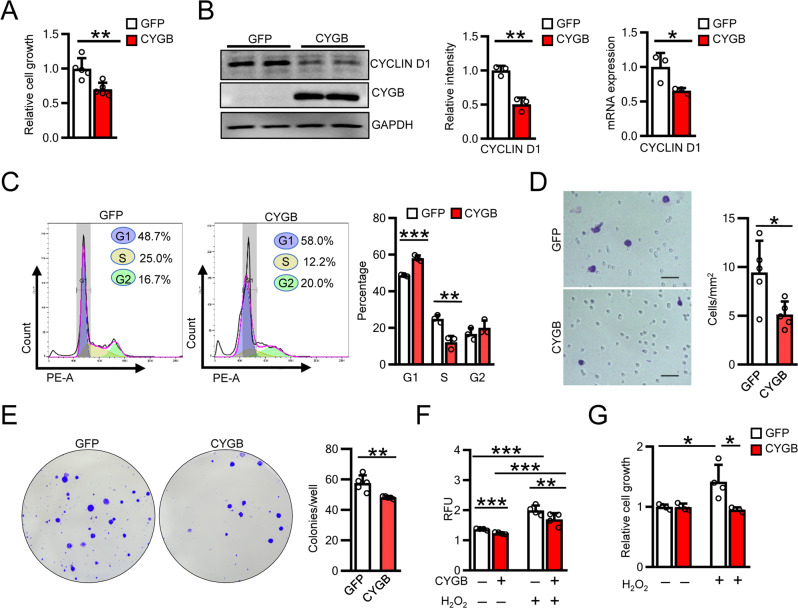
Overexpression of *CYGB* suppressed human pancreatic cancer cell proliferation. **A**, **B** Determination of MIA PaCa-2 cell proliferation by CCK-8 assay (**A**). The cells were stably transfected with lentiviral eGFP expression vector (GFP) or CYGB expression vector (CYGB), *n* = 5. Cell lysates were collected for immunoblotting (left and middle panels) and qRT-PCR analysis (right panel) for Cyclin D1, *n* = 3 (**B**). GAPDH was used as loading control. **C** Flow cytometric determination of cell cycle distribution in MIA PaCa-2 transfected with lentiviral eGFP expression vector (GFP) or CYGB expression vector (CYGB), *n* = 3. **D**, **E** Determination of cell migration for 24 h (**D**) and colony formation for 12 days (**E**) in MIA PaCa-2 transfected with lentiviral eGFP expression vector (GFP) or CYGB expression vector (CYGB), *n* = 3. Scale bars, 50 µm. **F** Determination of intracellular ROS by DCFDA assay using H_2_O_2_ 500 µM administration for 1 h in MIA PaCa-2 which were 48 h transiently transfected with empty vector (CYGB−) or pcDNA-6His-FLAG-CYGB vector (CYGB+), *n* = 4. **G** Determination of cell proliferation by CCK-8 assay in MIA PaCa-2 transfected with lentiviral eGFP expression vector (GFP) or CYGB expression vector (CYGB) under H_2_O_2_ 40 µM for 4 h, *n* = 4. Data were shown as mean ± SD from three independent experiments. *p < 0.05, **p < 0.01, ***p < 0.001, Student’s *t* test, two-tailed. RFU relative fluorescent units.

Finally, we examined the effect of stably overexpressing *Cygb* using the lentiviral vector on the function of MIA PaCa-2 cells. *Cygb* overexpression suppressed MIA PaCa-2 cell growth (Fig. 6A), downregulated cyclin D1 expression (Fig. 6B), and induced cell cycle arrest at the G1/S transition phase (Fig. 6C) compared with cells transfected with lentiviral eGFP vector. MIA PaCa-2 cell migration was reduced by CYGB overexpression (9.4 ± 3.2 for eGFP vector-transfected cells vs. 5.1 ± 1.1 for CYGB vector-transfected cells) (Fig. 6D). Colony formation assay showed similar results (Fig. 6E). Overexpression of *Cygb* in MIA PaCa-2 cells induced by 24 h of transient transfection (Supplemental Fig. 8B) diminished ROS production spontaneously and under stimulation by 500 µM H_2_O_2_ for 4 h (Fig. 6F) or 200 µM H_2_O_2_ for 1 h (Supplemental Fig. 8C). Whereas H_2_O_2_ (40 µM) stimulation for 4 h promoted MIA PaCa-2 cell proliferation, *Cygb* overexpression reversed this effect (Fig. 6G), indicating that the ROS scavenging effect of *Cygb* overexpression blunted MIA PaCa-2 cell proliferation, migration, and colony formation.

## Discussion

Recent evidence suggests that CYGB exerts an anti-carcinogenic effect in several human malignancies. Reduced *Cygb* expression and hypermethylation of the *Cygb* promoter are reported in patients with tylosis, non-small cell lung cancer, head and neck cancers, ovarian cancer, and breast cancer [54–62]. In particular, co-expression of CYGB and its potential upstream ∆Np63 negatively affected the survival of patients with early-stage non-small cell lung carcinoma [62]. In addition, low CYGB expression is found in glioma patients and is associated with higher histological grading and tumor recurrence [63]. In a CYGB-deficient mouse model, we found that 67% of mice aged 1–2 years spontaneously exhibit abnormalities and cancer development in multiple organs, including the liver, lungs, lymph nodes, and heart [35]. Furthermore, CYGB-deficient mice rapidly develop liver cancer in both chemically induced and high-fat diet models [37, 64]. Taken together, these findings indicate that CYGB serves a tumor suppressor function.

In the present study, we demonstrated the involvement of CYGB in PC pathophysiology. CYGB expression was observed in PSCs in both humans and mice but not in fibroblasts, endothelial, or ductal cells. Interestingly, CYGB-positive cells localized in areas surrounding carcinomas but not in the thick fibrotic septum, raising the question of whether activated PSCs migrate to the cancer area to support tumor growth. Indeed, activated PSCs are considered an important component of the cancer microenvironment that contributes to cancer progression and metastasis [14, 65]. Interactions between PSCs and PC cells have been intensively discussed [66, 67]. For instance, upon co-culture with conditioned medium from PSCs, BxPC3, and PANC-1 cancer cells exhibit increased proliferation and reduced apoptosis [53]. However, as shown in the present study, PSCs with enhanced CYGB expression trigger the suppression of MIA PaCa-2 proliferation, possibly through the secretion of CYGB. These results, together with our observations of a lower tumor incidence in TG mice and negative correlation between CYGB expression and PDAC tumor size in humans, indicate that CYGB-positive PSCs may suppress PC growth.

CYGB holds intrinsic O_2_^−^-binding capacity with the same affinity of heme iron for an exogenous ligand and the same equilibrium constant for O_2_ as myoglobin [33, 36]. In addition to its function as a gas carrier, CYGB acts as a cytoprotective molecule under hypoxia and oxidative stress [68–70]. Previously, we reported that TG mice are resistant to oxidative stress and fibrosis development in multiple models of liver fibrosis induced by thioacetamide, choline-deficient diet, or bile duct ligation [37, 48, 71]. By contrast, primary cultured HSCs isolated from CYGB-deficient mice show robust ROS accumulation, similar to those isolated from WT mice transfected with *Cygb* small interfering RNA [37]. In the present study, RNA-seq analysis revealed that rhCYGB treatment increased the expression of cellular antioxidant genes and decreased the expression of oxidative stress-related genes in MIA PaCa-2 cells, which showed reduced proliferation, colony formation, and migration. Together with the observed reduction of ROS-induced DNA damage in *Cygb*-overexpressing mouse PSCs in vivo as indicated by reduced 8-OHdG, these results demonstrate that CYGB serves a tumor suppressor function via its ROS scavenging ability.

*KRAS* mutations are frequently observed in PDAC, with 98% of mutations occurring at codon 12 of exon 2 in humans [72]. Furthermore, *KRAS* mutation-induced mitochondrial dysfunction is associated with accelerated ROS generation [73, 74]. To determine whether the effects of CYGB are dependent on *Kras* mutation, we performed direct sequencing of mouse *Kras* exon 1 at the position surrounding codon 12, 13 in untreated and DMBA-treated WT and TG mice and found no mutation (Supplemental Fig. 9). *KRAS* mutations at codon 12 were found in the MiaPaCa-1 and PANC-1 pancreatic cell lines but not in BxPC-3 cells or HPaSteCs (Supplementary Fig. 10A). We further examined the dose-dependent effects of rhCYGB in BxPC-3 cells and found that rhCYGB was able to inhibit BxPC-3 cell proliferation and the RNA expression of cell cycle–related genes (Supplementary Fig. 10B–C). Thus, the effects of CYGB appear to be independent of *KRAS* mutation.

We also wonder whether PSCs secrete factors other than CYGB that regulate the proliferation of cancer cells. Notably, neuroglobin, another globin family member, was reported to regulate the hypoxic response of neuronal cells through an Hif-1α- and Nrf2-mediated mechanism [75]. Thus, future studies examining PSC-derived factors would enable more in-depth exploration of the PSC deactivation and cancer suppression process.

In conclusion, we found that CYGB expression in PSCs was associated with reduced pancreatic tumor development and cancer cell proliferation, migration, and colony formation. These in vitro and in vivo findings confirm that CYGB plays an essential role in cellular homeostasis and cell division and suggest that targeting CYGB could open new therapeutic avenues for PC.

## Materials and methods

### Patients

We used paraffin-embedded blocks and tissue samples from 157 patients with PDAC who underwent surgical resection of primary PC at Osaka City University Hospital (Osaka, Japan). Patients were classified into well-differentiated, moderately differentiated, and poorly differentiated subgroups based on their tumor differentiation status. Clinical records and pathological reports were reviewed to obtain demographic data (i.e., age and gender), tumor-node-metastasis classification according to the 8th edition of the Union for International Cancer Control staging system [76], tumor marker values (carcinoembryonic antigen and carbohydrate antigen 19-9), and time from surgical resection to death (i.e., overall survival time) as shown in Supplemental Table 1. This study was approved by the ethics committee of Osaka City University, and all procedures were conducted in compliance with the Declaration of Helsinki. Written informed consent was obtained from all patients involved in the study.

### Animal experiments

*Cygb* with *mCherry* reporter-overexpressing TG mice were generated in our laboratory as previously described [71]. Mouse DMBA studies were conducted as previously described [77] with some modifications. Briefly, DMBA (TCI America, Portland, OR, USA) was used to induce PC in WT and TG male mice at 8–12 weeks of age. After mice were anesthetized by intraperitoneal injection of pentobarbital (Somnopentyl, Kyoritsu, Tokyo, Japan; 70 mg/kg body weight), 1.5 mg DMBA dissolved in 20 µL toluene (Wako, Osaka, Japan) was injected into the pancreatic tail. For sham treatment, the same volume of toluene was injected into the pancreatic tail. DMBA-treated WT and TG mice were randomly divided into three groups and sacrificed after 2 weeks, 2 months, or 3 months. In total, eight groups of mice were used, with 8–15 mice in each DMBA group and 3 mice in each sham group. No blinding method was used.

All animal protocols and experimental procedures were approved by the Institutional Animal Care and Use Committee of Osaka City University and were performed following the guidelines of the National Institutes of Health Guide for the Care and Use of Laboratory Animals. Mice were housed in a temperature-controlled (24 ± 1 °C) environment with a humidity of 55 ± 5%, alternating 12-h light/12-h dark cycle, and free access to water and standard rodent diet.

### Cell culture and treatment

HPaSteCs were purchased from ScienCell Research Laboratories (San Diego, CA, USA) and cultured in stellate cell medium (ScienCell Research Laboratories) supplemented with 2% fetal bovine serum (ScienCell Research Laboratories), 1% stellate cell growth supplement (ScienCell Research Laboratories), and 1% penicillin/streptomycin (ScienCell Research Laboratories) in a humidified atmosphere at 37 °C with 5% CO_2_.

MIA PaCa-2 and PANC-1 cells were acquired from American Tissue Cell Culture (ATCC; Manassas, VA, USA), and OCUP-A2 is a PC cell line established by our group from a patient with malignant pancreatic neoplasm and liver metastasis [78]. These cell lines were grown in Dulbecco’s Modified Eagle’s Medium (Gibco, Grand Island, NY, USA) supplemented with 10% heat-inactivated fetal bovine serum (Gibco) and 2.5% horse serum (ATCC; for MIA PaCa-2 cells only). BxPC-3 cells were acquired from the Japanese Collection of Research Bioresources Cell Bank (JCRB, Osaka, Japan) and cultured in RPMI 1640 medium with 10% heat-inactivated FBS (Gibco) in a humidified atmosphere at 37 °C with 5% CO_2_. Details of cell-based assays are provided in the Supplemental Materials.

### RNA-seq and data analysis

RNA-seq was performed on MIA PaCa-2 cells with or without rhCYGB treatment at a concentration of 4 µM (*n* = 3 per group). Total RNA was extracted and used for library preparation. RNA libraries were sequenced using the NovaSeq 6000 platform (Macrogen, Seoul, Korea). Further details are provided in the Supplemental Materials.

### Statistical analysis

All experiments were replicated at least three times. ImageJ was used to evaluate the band intensities for immunoblotting analysis (National Institutes of Health, Bethesda, MD, USA). Data are presented as dot plot graphs with median and interquartile range or bar graphs with mean and standard deviation. Statistical analysis was performed using F-tests for comparisons of variance; Student’s *t* tests (two-tailed), Mann–Whitney *U* tests, or Fisher’s exact tests for comparisons of two groups; and Kruskal–Wallis tests for comparisons of three or more groups. Significant differences are indicated as *p < 0.05, **p < 0.01, and ***p < 0.001. All statistical analyses were performed using GraphPad Prism version 8.0 (GraphPad Software, Inc., San Diego, CA, USA).

## Supplementary information

Supplemental Information

## Data Availability

All data supporting the findings of this study are available within the article and associated supplementary information files and from the corresponding author upon reasonable request. RNA-seq data were deposited in the GEO database under accession code GSE178358. Detailed description of the following techniques is available in the Supplemental Materials and Methods: (1) Histological, immunohistochemistry, and immunofluorescent analysis. (2) Recombinant human CYGB production. (3) Cell culture and treatment. (4) Vector construction. (5) Transwell migration assay. (6) Colony formation assay. (7) CCK8 assay. (8) In vitro distribution of rhCYGB. (9) Subcellular protein fractionation assay. (10) Cell cycle analysis. (11) Measurement of ROS. (12) RNA sequencing (RNA-seq) and data analysis. (13) Quantitative real-time PCR (qRT-PCR) assay. (14) Immunoblotting. (15) Direct sequencing analysis.
