# Supplementary material for: Cytoglobin attenuates pancreatic cancer growth via scavenging reactive oxygen species
Source: Oncogenesis. 2022 May 3;11(1):23. doi: 10.1038/s41389-022-00389-4 (PMC9065067; doi:10.1038/s41389-022-00389-4)
Supplement: Supplementary file 1 — Supplemental Information [file 41389_2022_389_MOESM1_ESM.pdf]

Cytoglobin Attenuates Pancreatic Cancer Growth via Scavenging Reactive Oxygen Species

Running title: Cytoglobin suppresses pancreatic cancer growth

Dinh Viet Hoang[1,2*], Le Thi Thanh Thuy[1*], Hoang Hai[1], Vu Ngoc Hieu[1], Kenjiro Kimura[3], Daisuke Oikawa[4], Yoshihiro Ikura[5], Ninh Quoc Dat[6], Truong Huu Hoang[1,7], Misako Sato-Matsubara[1], Minh Phuong Dong[1], Ngo Vinh Hanh[1], Sawako Uchida-Kobayashi[1], Fuminori Tokunaga[4], Shoji Kubo[3], Naoko Ohtani[8], Katsutoshi Yoishizato[9], and Norifumi Kawada[1†]

[1]Department of Hepatology, Graduate School of Medicine, Osaka City University, Osaka, Japan.

[2]Departmet of Anesthesiology, Cho Ray Hospital, Ho Chi Minh City, Vietnam.

[3]Hepato-Biliary-Pancreatic Surgery, Graduate School of Medicine, Osaka City University, Osaka, Japan.

[4]Department of Pathobiochemistry, Graduate School of Medicine, Osaka City University, Osaka, Japan.

[5]Department of Pathology, Takatsuki General Hospital, Takatsuki, Japan.

[6]Department of Pediatrics, Hanoi Medical University, Hanoi, Vietnam.

[7]Department of Pain Medicine and Palliative Care, Cancer Institute, 108 Military Central Hospital, Vietnam.

[8]Department of Pathophysiology, Graduate School of Medicine, Osaka City University, Osaka, Japan.

[9]Donated Laboratory for Synthetic Biology, Graduate School of Medicine, Osaka City University, Osaka, Japan.

[*] These authors shared co-first authorship.

[†]**Corresponding Author:**

Norifumi Kawada, M.D., Ph.D.

Department of Hepatology, Graduate School of Medicine, Osaka City University, 1-4-3 Asahimachi, Abeno, Osaka 545-8585, Japan.

Phone: +81-6-6645-3897; Fax: +81-6-6646-6072; E-mail: kawadanori@med.osaka-cu.ac.jp

Supplementary Materials and Methods

Animal Studies

Cygb with *mCherry* reporter-overexpressing (TG) mice were generated in our laboratory as previously described [1]. Briefly, in TG mice, exogenous *Cygb* was incorporated with a 2A-mCherry tag under the regulation of the *Cygb* promoter, which directs gene expression in target *Cygb*-expressing cells. In the pancreas, *Cygb* is overexpressed only in PSCs (Supplemental Fig.2). The founder TG mice were backcrossed with C57BL/6J wild-type mice for 3–6 generations, and only their offspring with 10 Cygb copies were used in this study. C57BL/6J WT mice were purchased from Japan SLC Inc. (Shizuoka, Japan).

All mice received humane care according to the Guide for the Care and Use of Laboratory Animals, National Institutes of Health. All protocols and experimental procedures were approved by the Institutional Animal Care and Use Committee of Osaka City University and were performed following the guidelines of the National Institutes of Health for the use of animals in research. Mice were housed in a temperature-controlled (24 ± 1°C) environment, with humidity levels of 55 ± 5%, alternating 12-hour light/12-hour dark cycles, and free access to water and a standard rodent diet.

Histological, immunohistochemistry, and immunofluorescence analysis

H&E, immunohistochemistry, and immunofluorescence analysis were performed as described previously [2]. The primary antibodies used are listed in Supplemental Table 2. Polyclonal antibodies against CYGB were generated in our laboratory [3]. For the quantification of pancreatic fibrosis, paraffin-embedded pancreas sections (5 μm) were stained with Picrosirius red (Sigma-Aldrich, Tokyo, Japan) and counterstained with Fast Green dye (Sigma-Aldrich)

(SiR-FG). Images were captured using the BZ-X700-All-in-One fluorescence microscope (Keyence Co., Osaka, Japan). The number of positively immune-stained cells were counted by taking 10 fields without overlapping at x 400 magnification per section. Percentages of positive areas per corresponding whole lobe of mouse pancreas were calculated using the BZ-X Analyzer software (Keyence Co.).

Recombinant human CYGB production

A 6His-tagged recombinant human CYGB (rhCYGB) was produced in our laboratory as previously reported [4]. Briefly, the open reading frame of human CYGB cDNA was cloned into the expression vector pRSET A (Thermo Fisher Scientific, Carlbad, CA, USA). CYGB, fused with 6His, was expressed in *Escherichia coli* BL-21AI (Thermo Fisher Scientific) and induced by 0.2% L-arabinose (Sigma-Aldrich). The rhCYGB protein was purified via immobilized metal affinity chromatography (HisTALON Superflow, Takara Bio, Mountain View, CA, USA) using fast protein liquid chromatography (ÄKTA pure, GE Healthcare Japan Corporation, Tokyo, Japan) and then polished by size exclusion chromatography (HiLoad 16/600 Superdex 75 prep grade, GE Healthcare). rhCYGB was subjected to 12.5% sodium dodecyl sulfate-polyacrylamide gel electrophoresis (SDS-PAGE) to verify the purity, which finally confirmed no toxicity *in vitro*, no endotoxin contamination, and typical heme activity in rhCYGB.

Cell culture and treatment

Human PSCs (HPaSteCs) were purchased from ScienCell Research Laboratories (San Diego, CA, USA) and cultured in Stellate Cell Medium (SteCM, ScienCell Research Laboratories), supplemented with 2% fetal bovine serum (FBS, ScienCell Research Laboratories), 1% stellate cell growth supplement (SteCGS, ScienCell Research Laboratories), and 1%

penicillin/streptomycin (P/S, ScienCell Research Laboratories) in a humidified atmosphere at 37°C and 5% CO_2. HPaSteCs were passaged when sub-confluent and used between passages 3–10 for experiments. We confirmed that HPaSteCs are not an immortalized cell line but are human normal diploid PSCs because they become senescent after 15 population doublings under the recommended culture conditions with SteCM, 2% FBS and associated supplement solution (thereafter called, S+).

MIA PaCa-2 and PANC-1 were acquired from American Tissue Cell Culture (ATCC, Manassas, VA, USA), OCUP-A2 was established as a pancreatic cancer cell line in our university from a malignant pancreatic neoplasm with liver metastasis patient in a man in his 30s [5]. These cell lines were grown in Dulbecco's Modified Eagle's Medium (DMEM, Gibco, Grand Island, NY, USA) supplemented with 10% heat-inactivated FBS (Gibco) and 2.5% horse serum (for MIA PaCa-2 only) (ATCC) BxPC-3 were acquired from Japanese Collection of Research Bioresources Cell Bank (JCRB, Osaka, Japan) and cultured in RPMI 1640 medium with 10% heat-inactivated FBS (Gibco) in a humidified atmosphere at 37°C and 5% CO_2.

Cells were seeded into six-well plates at 2×10^5 cells/well, 12-well plates at 1×10^5 cells/well, 24-well plates at 0.5×10^5 cells/well, and 96-well plates (Greiner Bio-One, Tokyo, Japan) at 5,000 cells/well in a final volume of 2, 1, 0.5, and 0.1 mL of the appropriate medium, respectively. On the following day, for the HPaSteCs experiments, the medium was replaced with 0.2% FBS/SteCM/S- medium to activate PSCs, cells were stimulated with 4 ng/ml recombinant human basic fibroblast growth factor 2 (FGF2, Wako, Tokyo, Japan) for 48 hours. For experiments using MIA PaCa-2, PANC-1, OCUP-A2, and BxPC-3 the medium was changed to 1% FBS/DMEM, then 24 hours later, rhCYGB were added in a dose-dependent manner. MIA PaCa-2 was challenged with N-Acetyl-L-Cysteine (Sigma-Aldrich) and Glutathione (Sigma-Aldrich) by adding to medium culture in a dose-dependent manner for 48 hours, then used for determination of cell proliferation or qRT-PCR analysis.

Vector construction

The open reading frame of human CYGB cDNAs were amplified by reverse transcription-PCR. The cDNAs were ligated to the appropriate epitope sequences and cloned into the pcDNA3.2 (Invitrogen, Carlbad, CA, USA) vector with 6His and Flag tags (pcDNA3.2-His-FLAG-CYGB). For lentiviral transduction, pCSII-CMVRfA-IRES-Blast (RIKEN BioResource Research Center, Ibaraki, Japan) was used. For stable expression of His-FLAG-tagged CYGB or control vector His-FLAG-tagged enhanced green fluorescent protein (eGFP) in HPaSteCs or MIA PaCa-2 cells, lentiviral infection followed by selection with 5 μg/ml blasticidin was performed. The pcDNA3.2-His-FLAG-CYGB and empty vector were used only for transient transfection of MIA PaCa-2 cells in Bes-H_2O_2-Ac assay (see below) since this Bes-H_2O_2 probe for cell-derived H_2O_2 was measured at excitation wavelength 485 nm which interferes with eGFP signal in lentiviral vector.

Transwell migration assay

One hundred μL of MIA PaCa-2 cell solution (2×10^4 cells) was plated in a transwell insert 8.0-μm pore size (BD Falcon, Bedford, MA, USA), incubated for 10 minutes at 37 °C and 5% CO_2 to allow the cells to settle down, then very carefully added 600 μL of DMEM into the bottom of the lower chamber in a 24 well-plate (Corning Inc., Corning, NY, USA). The plate was then incubated for 24 hours at 37°C and 5% CO_2. At the end of the assay, the cells that had migrated to the other side of the membrane were fixed with 4% paraformaldehyde at room temperature for 30 minutes and stained with 0.1% crystal violet solution for 30 min. The number of cells in each field was counted after taking pictures using the BZ-X700-All-in-One fluorescence microscope (Keyence Co.).

Colony formation assay

MIA PaCa-2 cells were plated onto 12-well plates (100 cells/well) in 1 mL of DMEM containing 10% FBS and 2.5% horse serum for colony formation. After 12 – 14 days, colonies that were formed were fixed with 10% methanol for 20 minutes and stained with 0.5 % crystal violet solution for 15 min. Crystal violet was then carefully removed, and the cells were washed with PBS 2 times. The plates with colonies were allowed to dry in normal air at room temperature. The number of clones was counted under a light microscope.

CCK8 assay

MIA PaCa-2 cells were seeded in a 96-well plate at 1×10^4 cells/well and incubated in 5% CO_2 at 37°C for 24 hours. Then, the medium was changed to DMEM supplemented with 1% FBS, and the following day, cells were treated with rhCYGB in a time- and dose-dependent manner. To measure the cell viability of MIA PaCa-2 cells, we used the Cell Counting Kit-8 (CCK8, Dojindo Laboratories, Kumamoto, Japan) following the manufacturer’s protocol. Absorbance was measured at 450 nm using microplate reader Varioskan lux (Thermo Fisher Scientific).

***In vitro* distribution of rhCYGB**

RhCYGB protein was labeled with Alexa tetrafluorophenyl esters 488 (molecular weight = 643 g/mole; peak excitation at 495 nm; peak emission at 519 nm), which were obtained from Molecular Probes (Eugene, OR, USA), as previously reported [4]. The translocation of Alexa-rhCYGB into cells was determined using the following experiments. MIA Paca-2 were seeded on 8-well chamber slides (Thermo Fisher Scientific) at 5,000 cells/well in a final volume of 250 µL complete DMEM and incubated for 24 hours, at 37°C, 5% CO2. After changing to fresh medium, Alexa-rhCYGB (10 µg/mL) was added and incubated for 24 hours. Hoechst

33258 (Thermo Fisher Scientific), a nucleus staining dye, was added to cells for the last 10 minutes. The cells were then washed three times with PBS, intracellular localization of Alexa-rhCYGB was observed with a Zeiss LSM 800 confocal microscope (Carl Zeiss Microscopy, Jena, German).

Subcellular protein fractionation assay

To evaluate the intracellular localization of rhCYGB, we used a Subcellular Protein Fractionation Kit for cultured cells (Thermo Scientific). MIA PaCa-2 were seeded in complete medium at 1.5×10^6 1 cells in a 10 cm dish for one day, and the medium was replaced with fresh 1% FBS/DMBA the following day. Then, cells were treated with 40 μg/mL rhCYGB for 24 hours before performing cellular fractionation to separate cytoplasmic, membrane, nuclear soluble, chromatin-bound, and cytoskeletal protein extracts according to the manufacturer's protocol.

Cell cycle analysis

For cell cycle analysis, cells were plated onto 6-well plates at 3×10^5 cells/well. After serum starvation overnight and treatment with different conditions, cells were trypsinized and collected by centrifugation (1500 g, 5 min), followed by washing two times with PBS and fixed in 70% ethanol at 4°C for 24 h. After washing with PBS, the cells were stained with propidium iodide in the staining solution supplemented with RNase A (Abcam, Cambridge, UK) for 30 min at 37°C. The cell cycle was assessed using the BD LSR II flow cytometer (BD Biosciences, CA, USA) and quantified using FlowJo software (BD Biosciences).

Measurement of ROS

BES-H_2O_2-Ac assay: The hydrogen peroxidase scavenging activity was evaluated by BES-H_2O_2-Ac assay (Wako). Briefly, MIA PaCa-2 were plated onto 96-well plate at 1×10^4 cells/well and precultured as describe above. Cells were pre-treated with or without rhCYGB 2.5 µM for 24 hours; or transiently transfected with pcDNA3.2-His-FLAG-CYGB or empty vector 24 hours before incubated with BES-H_2O_2-Ac 2 µM for 1 hour and then stimulated with H_2O_2 500 µM for 1 hour. The cells were washed twice with PBS and fluorescence signal was measured as relative fluorescent units (RFU) at excitation wavelength 485 nm and emission wavelength 530 nm by spectrophotometer (Thermo Fisher Scientific).

DCFDA assay: Intracellular ROS production was measured with the DCFDA-Cellular ROS Detection Assay Kit (Cat #ab113851, Abcam). MIA PaCa-2 were seeded on 96-well plates as described above. Cells were pre-treated with or without rhCYGB 2.5 µM for 24 hours; or transiently transfected with pcDNA3.2-His-FLAG-CYGB or empty vector for 24 hours before being stimulated with H2O2 500 µM for 4 hours. Then, the cells were washed by PBS 2 times before stained with 20 µM ROS-sensitive fluorescent dye (DCFDA) at 37°C to assess the ROS levels. After 45 minutes of incubation, the cells were washed twice with PBS, and the fluorescence signal was measured as relative fluorescent units (RFU) at excitation wavelength 485 nm and emission wavelength 530 nm by spectrophotometer (Thermo Fisher Scientific).

RNA sequencing (RNA-seq) and data analysis

RNA-Seq was performed on MIA PaCa-2 with or without rhCYGB treatment at the concentration of 4 µM (n = 3 each group). Total RNA was extracted using RTL buffer and purified using the RNeasy Mini Kit (Qiagen, Valencia, CA, USA) according to the manufacturer's protocol. After eliminating DNA contamination using DNase, TruSeq Stranded

mRNA Sample Prep Kit was used for the library prep process. The resulting mRNA was fragmented and reverse-transcribed using random primers into cDNA, and adapters were ligated onto both ends of the cDNA fragments. RNA libraries were then sequenced using the NovaSeq 6000 platform (Macrogen, Seoul, Korea). Quality control of the sequenced raw reads was analyzed using sequencing control software, FastQC v0.11.7. Trimmed reads were mapped to the reference genome with HISAT2, and then the transcript was assembled by StringTie, with aligned reads. Expression profiles are represented as reading counts and normalization values, which are based on the transcript length and coverage depth. The Fragments Per Kilobase of transcript per Million mapped reads value or the Reads Per Kilobase of transcript per Million mapped reads is used as the normalization value.

Quantitative real-time PCR (qRT-PCR) assay

Total RNA was extracted from the cells or pancreas tissues using the RNeasy Mini Kit (Qiagen) and reverse-transcribed to cDNA by oligo (dT)12–18 Primer (ReverTra Ace, Toyobo, Osaka, Japan) according to the manufacturer's instruction. Gene expression was measured by qRT-PCR using cDNA, THUNDERBIRD SYBR qPCR Mix reagent (Toyobo), and a set of gene-specific oligonucleotide primers and probes (Supplemental Tables 4 and 5) using the Applied Biosystems Prism Fast 7500 system (Applied Biosystems, Tokyo, Japan). Glyceraldehyde-3-phosphate dehydrogenase (GAPDH) level was used to normalize relative mRNA abundance.

Immunoblotting

Proteins isolated from the tissues (30 μg) or cells (6 – 10 μg) were subjected to SDS-PAGE and transferred to Immuno-Blot® PVDF membranes (Bio-Rad, Hercules, CA, USA). After blocking with 5% skim milk, the membranes were incubated with primary antibodies overnight

at 4°C (Supplemental Table 3) and then labeled with horseradish peroxidase-conjugated secondary antibodies (Dako, Agilent Technologies, Santa Clara, CA, USA). Immunoreactive bands were visualized by enhanced chemiluminescence using the L-012 substrate (ImmunoStar LD, Wako, Osaka, Japan) and documented with the Fujifilm Image Reader LAS-3000 (Fujifilm, Tokyo, Japan) coupled to an image analysis software (Multi-Gauge, Fujifilm). GAPDH was used as the loading control.

Direct sequencing analysis

We examined the mouse Kras mutation at codon 12, 13 in DNA samples isolated from pancreatic tissue of control and DMBA treated mice. Genomic DNA was extracted from the pancreas using DNeasy Blood & Tissue Kits (QIAGEN, Hilden, Germany). A fragment of human KRAS exon 2 was amplified via polymerase chain reaction (PCR) using the following primers: forward, 5′-AAGGCCTGCTGAAAATGAC-3′; reverse, 5′-TGGTCCTGCACCAGTAATATG-3′ [6]; mouse Kras exon 1 primers: forward, 5′-CTTTACAAGCGCACGCAGAC-3′; reverse, 5′-AGGTTACTCTGTACATCTGTAGTCA-3′; PCR was performed in a total volume of 20 µL with 1 × Premix Ex Tag (TaKaRa Bio Inc., Shiga, Japan), 300 nM of each primer and 100 ng of genomic DNA. The PCR protocol was performed at 94 °C for 2 min followed by 35 cycles of 98 °C for 10 s, 58 °C for 30 s and 68 °C for 30 s, with a final extension at 68 °C for 7 min. PCR products were sequenced bi-directionally using a BigDye Terminator v3.1 Cycle Sequencing Kit and an 3130XL Genetic Analyser (Applied Biosystems). Mus musculus chromosome 6 clone RP23-188E5 strain C57BL6/J, AC019026.12, was used as reference sequence.

Data availability

All data supporting the findings of this study are available within the article and associated supplementary information files and from the corresponding author upon reasonable request. RNA-seq data were deposited in the GEO database under accession code GSE178358.

References

1. Thi Thanh Hai, N., et al. Selective overexpression of cytoglobin in stellate cells attenuates thioacetamide-induced liver fibrosis in mice. Sci Rep 8, 17860 (2018).
2. Thuy le, T. T., et al. Promotion of liver and lung tumorigenesis in DEN-treated cytoglobin-deficient mice. Am J Pathol 179, 1050-1060 (2011).
3. Kawada, N., et al. Characterization of a stellate cell activation-associated protein (STAP) with peroxidase activity found in rat hepatic stellate cells. J Biol Chem 276, 25318-25323 (2001).
4. Dat, N. Q., et al. Hexa Histidine-Tagged Recombinant Human Cytoglobin Deactivates Hepatic Stellate Cells and Inhibits Liver Fibrosis by Scavenging Reactive Oxygen Species. Hepatology 73, 2527-2545 (2021).
5. Miura, K., et al. Establishment and characterization of new cell lines of anaplastic pancreatic cancer, which is a rare malignancy: OCUP-A1 and OCUP-A2. BMC cancer 16, 268-268 (2016).
6. Franklin, W. A., et al. KRAS mutation: comparison of testing methods and tissue sampling techniques in colon cancer. J Mol Diag: JMD 12, 43-50 (2010).

Supplemental Table 1. Characteristics of PDAC patients

Characteristics	Value (n=157)
Age, median years [IQR*]	70 (63-75)
Gender (male/female)	76/81
Histological differentiation	
Well, n (%)	22 (14.0)
Moderate, n (%)	114 (72.6)
Poor, n (%)	21 (13.4)
TNM stage (%)	
IA, n (%)	7 (4.45)
IB, n (%)	14 (8.9)
IIA, n (%)	56 (35.7)
IIB, n (%)	63 (40.1)
III, n (%)	6 (3.8)
IV, n (%)	11 (7.0)
TNM – T stage	
1, n (%)	8 (5.1)
2, n (%)	24 (15.3)
3, n (%)	120 (76.4)
4, n (%)	5 (3.2)
TNM – N stage = 1 n (%)	74 (47.1)
TNM – M stage = 1 n (%)	11 (7.0)
CA19.9, median [IQR*]	131.00 [33.75, 717.00]
CEA, median [IQR*]	4.00 [2.40, 6.30]
Survival days, median [IQR*]	564 [305, 1167]

IQR*, inter quarter range

Supplemental Table 2. List of antibodies used for immunohistochemical and immunofluorescent staining in this study

Name	Origin	Clonality	Source	Dilution
Anti-human CYGB	Rabbit	Polyclonal	Our laboratory	1/100
Anti-mouse CYGB	Rabbit	Polyclonal	Our laboratory	1/300
Anti-αSMA (Clone 1A4)	Mouse	Monoclonal	Dako (M0851)	1/200
Anti-human and mouse CD68	Rabbit	Polyclonal	Abcam (ab125212)	1/100
Anti-human CD68	Mouse	Monoclonal	Dako (M0876)	1/200
Anti-Vimentin	Rat	Monoclonal	R&D systems (MAB2105)	1/100
Anti-S100A4	Mouse	Monoclonal	Invitrogen (MA5-31332)	1/100
Anti-Desmin	Goat	Polyclonal	Santa Cruz (sc-7559)	1/100
Anti-CD31	Goat	Polyclonal	R&D systems (AF3628)	1/100
Anti-Cytokeratin 19 (M-17)	Goat	Polyclonal	Santa Cruz (sc-33111)	1/100
Anti-AE1/AE3	Mouse	Monoclonal	Dako (IS053)	1/100
Anti-Fibulin 2	Mouse	Monoclonal	Santa Cruz (sc-2714830)	1/100
Anti-P53	Rabbit	Polyclonal	Santa Cruz (sc-6243)	1/100
Anti-53BP1	Rabbit	Polyclonal	Bethyl Laboratories (A300-272A)	1/2000
Anti-NRF2	Rabbit	Polyclonal	Abcam (ab137550)	1/500
Anti-IL6	Rabbit	Polyclonal	Abcam (ab6672)	1/100
Anti-Ki67	Rabbit	Polyclonal	Abcam (ab16667)	1/100
Anti-mCherry	Mouse	Monoclonal	Abcam (ab125096)	1/100

Supplemental Table 3. List of antibodies used for immunoblotting analysis in this study

Name	Origin	Clonality	Source	Dilution
Anti-human CYGB	Rabbit	Polyclonal	Our laboratory	1/1000
Anti-mouse CYGB	Rabbit	Polyclonal	Our laboratory	1/1000
Anti-αSMA (Clone 1A4)	Mouse	Monoclonal	Dako (M0851)	1/3000
Anti-Collagen Type I	Rabbit	Polyclonal	Abcam (ab292)	1/1000
Anti-GAPDH (Clone 6C5)	Mouse	Monoclonal	Millipore (MAB374)	1/10000
Anti-Cyclin D1	Rabbit	Monoclonal	Cell Signaling (#55506)	1/1000
Anti-Cyclin E2	Rabbit	Polyclonal	Cell Signaling (#4132)	1/1000
Anti-P27	Mouse	Monoclonal	Cell Signaling (#3698)	1/1000
Anti-Phospho-ERK	Rabbit	Monoclonal	Cell Signaling (#4370)	1/1000
Anti-ERK	Rabbit	Monoclonal	Cell Signaling (#4695)	1/1000
Anti-β-Actin	Rabbit	Polyclonal	MBL (PM053)	1/10000
Anti-Lamin A	Mouse	Monoclonal	Santa Cruz (sc-71481)	1/1000
Anti-N-Cadherin	Rabbit	Polyclonal	Abcam (ab18203)	1/1000

Supplemental Table 4. List of human qRT-PCR primers used in this study

Gene	Sequence
GAPDH	Forward GCACCGTCAAGGCTGAGAAC<br>Reverse TGGTGAAGACGCCAGTGGA
CYGB	Forward CGAGATGGAGATCGAGCG<br>Reverse CGAGGGGAAGTTCACAAAGA
PDGFRB	Forward CCCTTATCATCCTCATCATGC<br>Reverse CCTTCCATCGGATCTCGTAA
αSMA	Forward CAGCCAAGCACTGTCAGG<br>Reverse CCAGAGCCATTGTCACACAC
COL1A1	Forward AAGAGGAAGGCCAAGTCGAG<br>Reverse CACACGTCTCGGTCATGGTA
COL3A1	Forward CTGGACCCCAGGGTCTTC<br>Reverse CATCTGATCCAGGGTTTCCA
SOD1	Forward GGTGGGCCAAAGGATGAAGAG<br>Reverse CCACAAGCCAAACGACTTCC
HMOX1	Forward AAGACTGCGTTCCTGCTCAAC<br>Reverse AAAGCCCTACAGCAACTGTCG
HMOX2	Forward TCAGCGGAAGTGGAAACCTC<br>Reverse AGAAGTCCTTGACAAACTGGGT
HSPA1A	Forward CAATTTCCTGTGTTTGCAATGTTGAAATTT<br>Reverse TGCATGTAGAAACCGGAAAAAA
CDKN1A (P21)	Forward AGCAGAGGAAGACCATGTGGA<br>Reverse GGAGTGGTAGAAATCTGTCATGCT
CDKN1B (P27)	Forward AAGGGCCAACAGAACAGAAG<br>Reverse GGATGTCCATTCAATGGAGTC
CYCLIN D1	Forward GCTGCGAAGTGGAAACCATC<br>Reverse CCTCCTTCTGCACACATTTGAA
CYCLIN E2	Forward TCAAGACGAAGTAGCCGTTTAC<br>Reverse TGACATCCTGGGTAGTTTTCCTC
GADD45G	Forward CAGATCCATTTTACGCTGATCCA<br>Reverse TCCTCGCAAAACAGGCTGAG
CDK2	Forward CCAGGAGTTACTTCTATGCCTGA<br>Reverse TTCATCCAGGGGAGGTACAAC
CDK4	Forward ATGGCTACCTCTCGATATGAGC<br>Reverse CATTGGGGACTCTCACACTCT
E2F1	Forward CATCCCAGGAGGTCACTTCTG<br>Reverse GACAACAGCGGTTCTTGCTC

Supplemental Table 5. List of mouse qRT-PCR primers used in this study

Gene	Sequence
Gapdh	Forward TGCACCACCAACTGCTTAG<br>Reverse GGATGCAGGGATGATGTTC
Col1a1	Forward GAGCGGAGAGTACTGGATCG<br>Reverse GTTCGGGCTGATGTACCAGT
Timp1	Forward ACTCGGACCTGGTCATAAGGGC<br>Reverse TTCCGTGGCAGGCAAGCAAAGT
CD68	Forward GGGGCTCTTGGGAACTACAC<br>Reverse GTACCGTCACAACCTCCCTG
Il6	Forward CCGCTATGAAGTTCCTCTCTGC<br>Reverse ATCCTCTGTGAAGTCTCCTCTCC
Tnfα	Forward CTCTTCTCATTCCTGCTTGTGG<br>Reverse AATCGGCTGACGGTGTGG
Cxcl9	Forward ATTGTGTCTCAGAGATGGTGCTAATG<br>Reverse TGAAATCCCATGGTCTCGAAAG
Ccl3	Forward TTCTCTGTACCATGACACTCTGC<br>Reverse CGTGGAATCTTCCGGCTGTAG
Ccl5	Forward GCTGCTTTGCCTACCTCTCC<br>Reverse TCGAGTGACAAACACGACTGC
Il1β	Forward ACTCCTTAGTCCTCGGCCA<br>Reverse TGGTTTCTTGTGACCCTGAGC
Cat	Forward AGCGACCAGATGAAGCAGTG<br>Reverse TCCGCTCTCTGTCAAAGTGTG
Sod1	Forward ACCAGTGCAGGACCTCATTTTAA<br>Reverse TCTCCAACATGCCTCTCTTCATC

Supplemental Figure 1

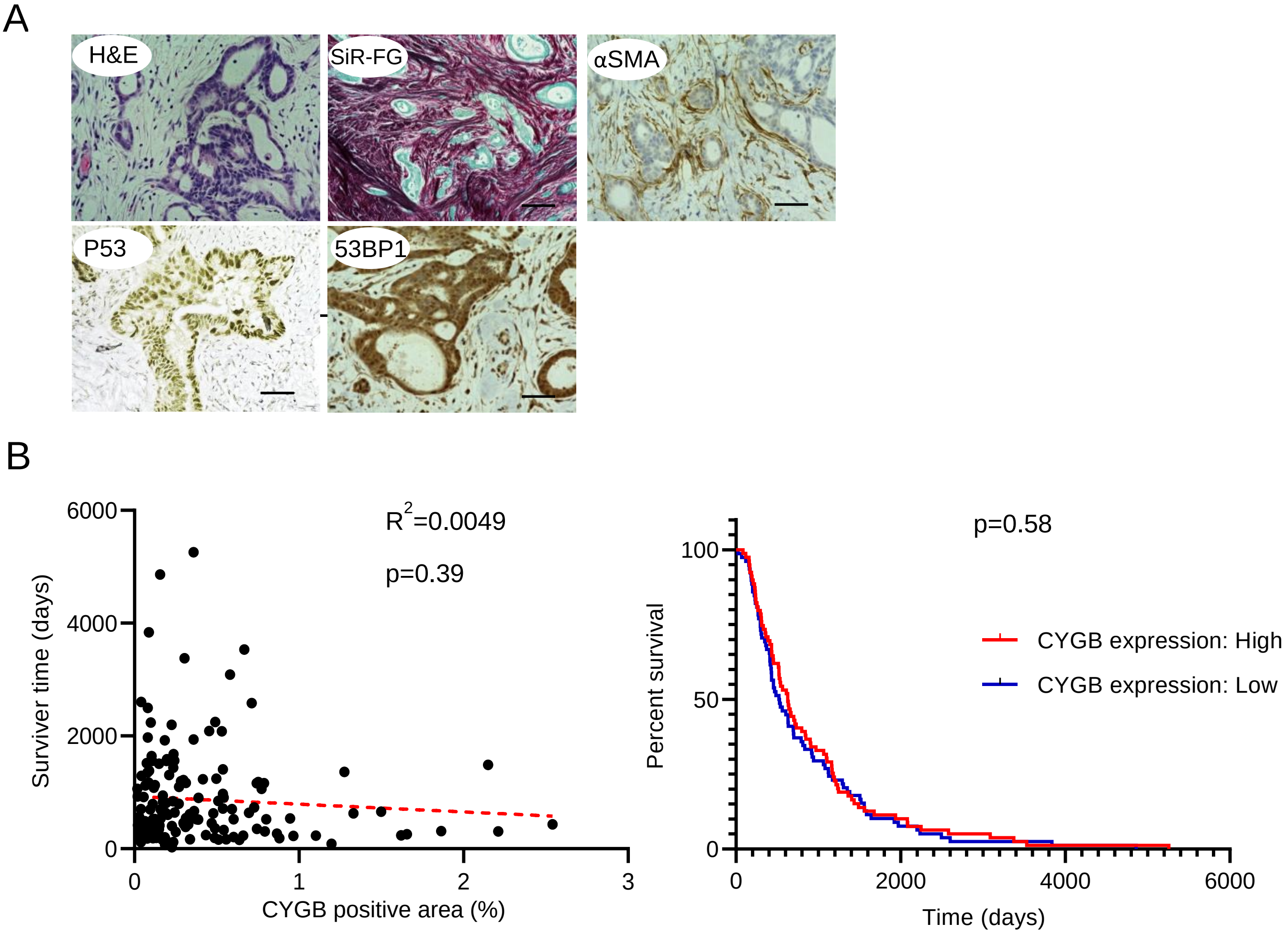

Supplemental Figure 1

Characterization of human pancreatic ductal adenocarcinoma

(A) Representative human pancreatic cancer tissue microscopic images of HE, SiR-FG and immunohistochemical staining for αSMA, TP53 and 53BP1, hematoxylin was used to visualize nuclei. Scale bars 20 µm. (B) Correlations between the percentage of CYGB positive area and overall survival in 157 patients with PDAC (left panel) and Kaplan-Meier analysis (right panel) to compare overall survival between two groups. Receiver operating characteristic analysis was used to determine the most appropriate cut-off value. The percentage of CYGB positive area ≤ 0.154% refers to low CYGB expression.

Supplemental Figure 2

A

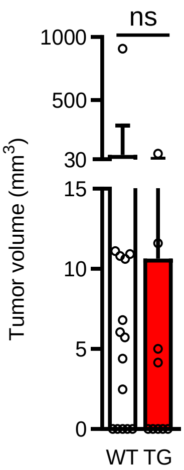

B

DMBA 3M	WT	TG
Numbers of mice at starting point	20	12
Number of death mice during observation period	5	3
Numbers of mice at terminal time	15	9
Adenocarcinoma pattern	12	2
Mixed of adenocarcinoma and sarcomatoid-like carcinoma	2	3
Number of non-tumor bearing mice	1	4

C

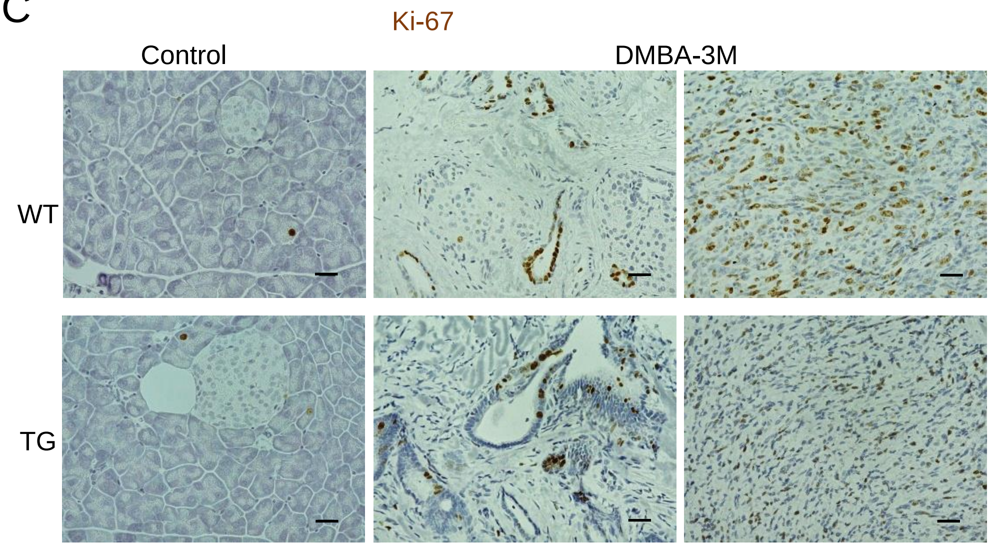

D

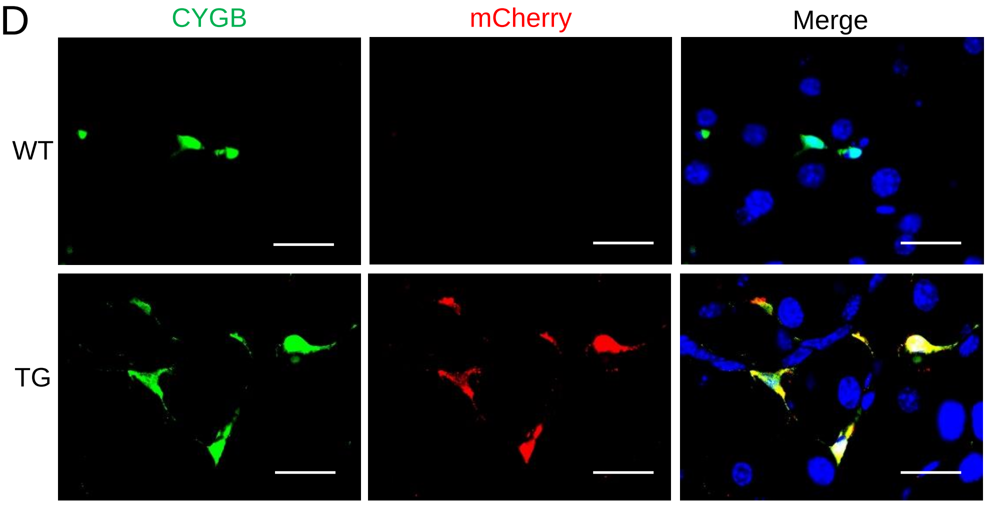

Supplemental Figure 2 (continue)

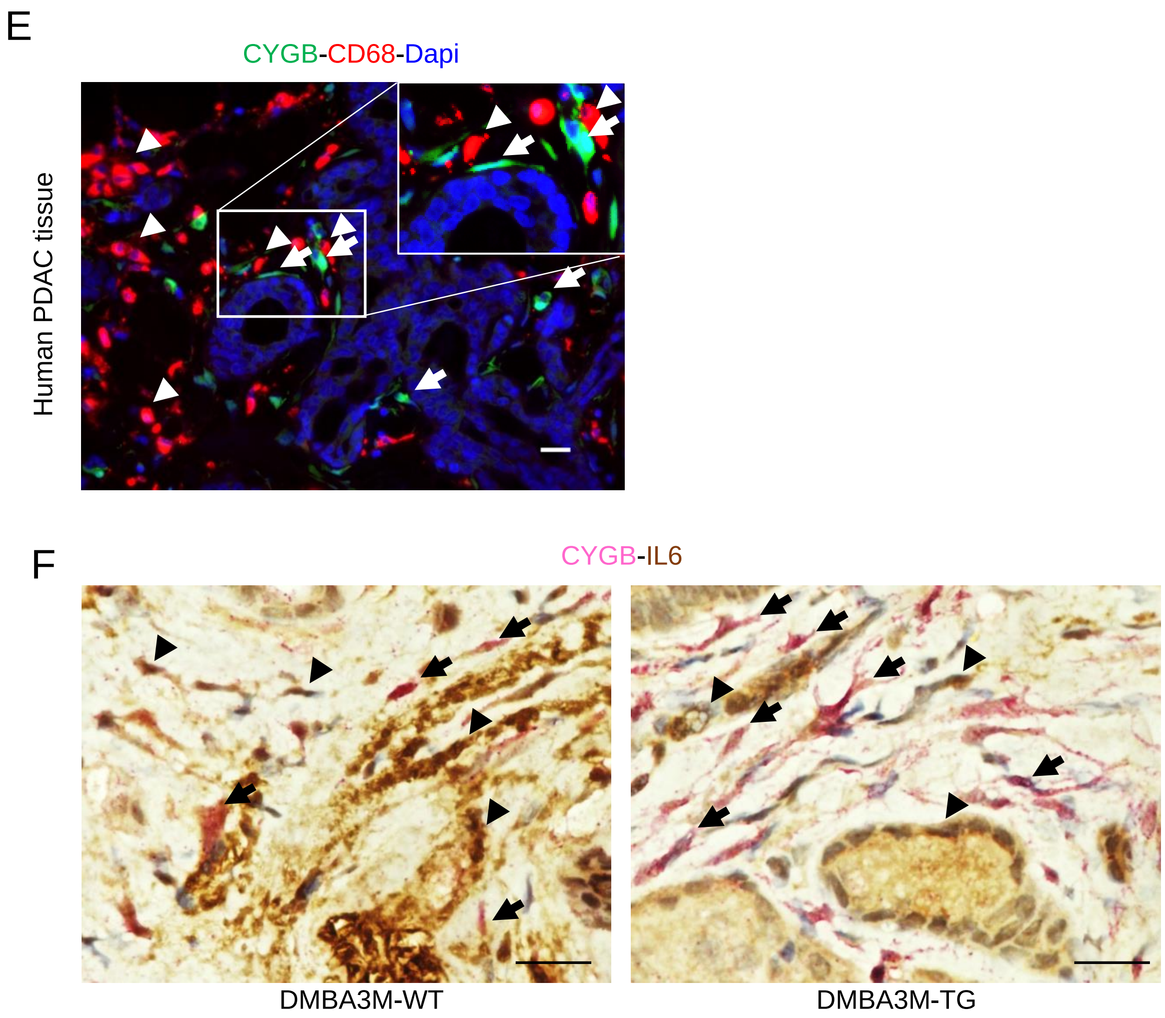

Supplemental Figure 2

Characteristics of pancreas tissues from WT and TG mice treated with DMBA for 3 months

(A - B) Tumor volume (A) and histological findings (B) of DMBA treated mice for 3 months (DMBA 3M). (C) Representative pancreatic tissue microscopic images of IHC staining for Ki-67 in control and DMBA-3M treated mice. (D) Representative normal wild-type (WT) and CYGB transgenic (TG) mouse pancreatic tissue microscopic images of IF staining for cytoglobin (green) and mCherry CYGB reporter (red). (E) Double immunofluorescent detection of CYGB (green) with macrophage makers–CD68 (red) in human pancreatic cancer tissues showed no co-localization of these proteins. Arrows, CYGB single positive cells; arrowhead, CD68 single positive cells. (F) Representative mouse pancreatic cancer tissue microscopic images of double IHC staining for CYGB (pink) and IL-6 (brown) in control and DMBA-3M treated mice. Arrows, CYGB single positive cells; arrowhead, IL6 single positive cells. Hematoxylin (C, F panels) and 4′ ,6-diamidino-2-phenylindole (Dapi, D, E panels) were used to visualize nuclei.

Supplemental Figure 3

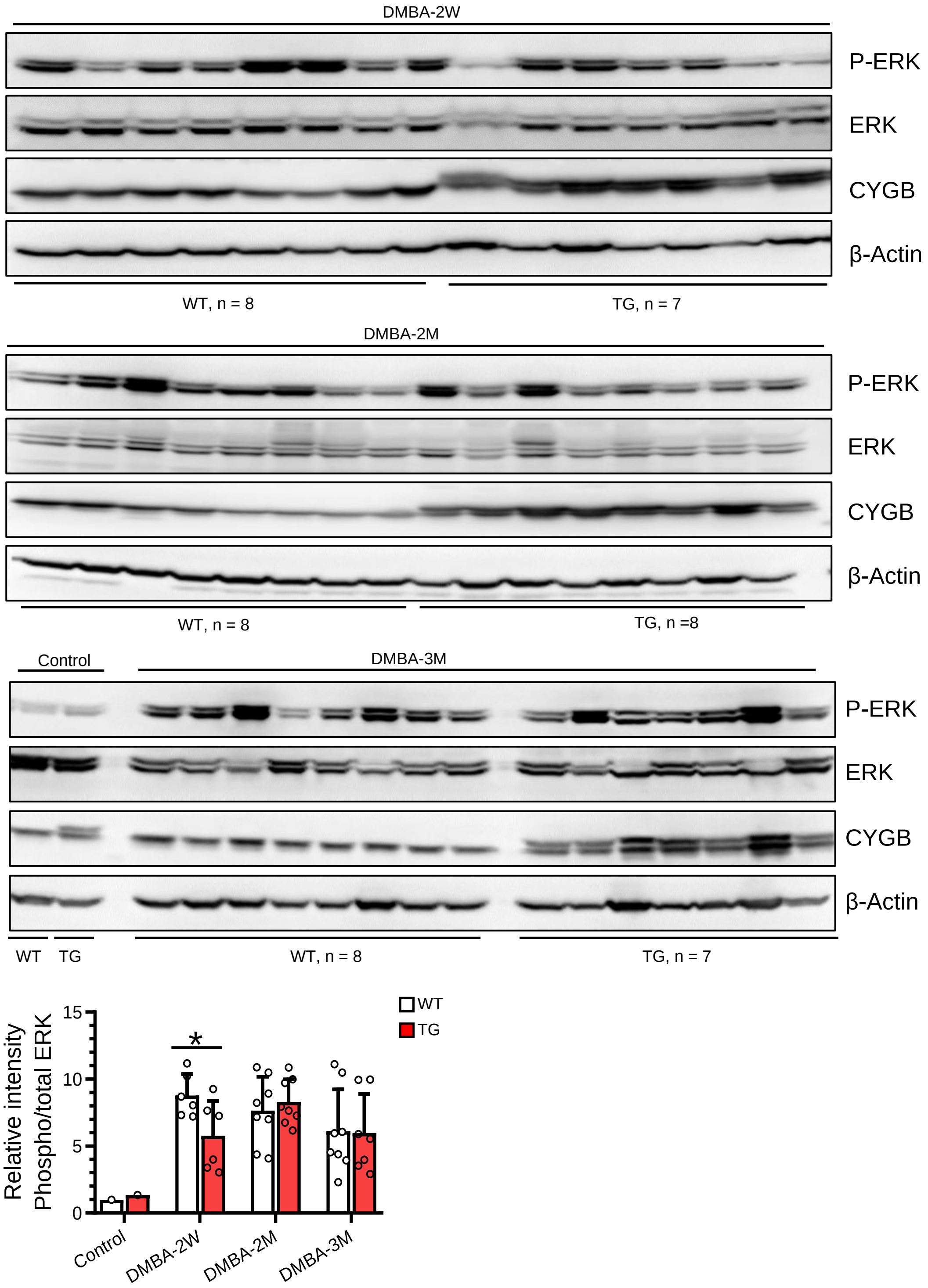

Supplemental Figure 3

Time-dependent expression of phospho-ERK and total ERK proteins in DMBA treated WT and TG mice

Immunoblotting analysis and their quantification of phospho-ERK and total ERK proteins in DMBA treated WT and TG mice time dependently. β-Actin was used as loading control. *$p < 0.05$, Student's t-test.

Supplemental Figure 4

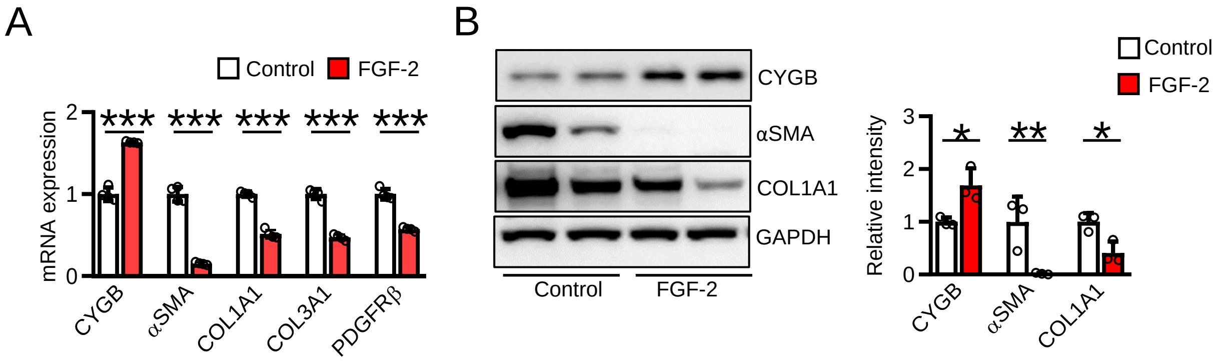

Supplemental Figure 4

Effect of FGF2 on CYGB, αSMA and Collagen expression in HPaSteCs

(A-B) qRT-PCR analysis of CYGB, αSMA, COL1A1, COL3A1, and PDGFRβ (A), and immunoblotting analysis of CYGB, COL1A1 and αSMA along with their quantifications (B) of HPaSteCs treated without (control) or with 4 ng/mL of FGF-2 for 48 hours. Data were shown as mean ± SD from three independent experiments n=4 each group. **p* < 0.05, ***p* < 0.01, ****p* < 0.001, Student's t-test.

Supplemental Figure 5

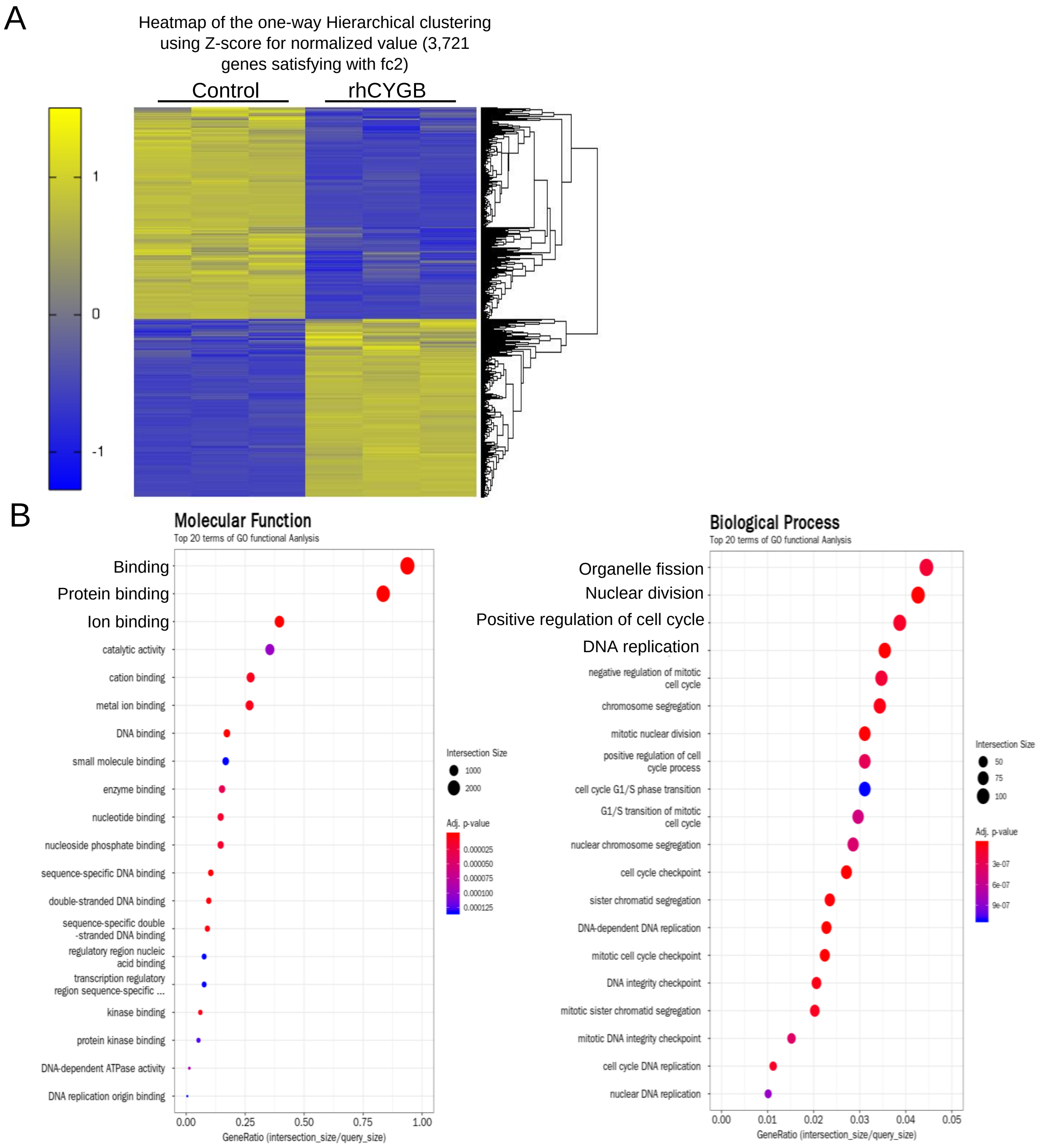

Supplemental Figure 5

RNA seq analysis of MIA PaCa-2 treated with rhCYGB

(A) Heatmap analysis of genes in MIA PaCa-2 treated with rhCYGB (4 µM, 48 h) that were 2-fold changed significantly compared to untreated controls by RNA-Seq (n = 3). (B) Top 20 terms of GO functional analyzed differentially expressed genes with gProfiler for gene set enrichment analysis with regard to molecular function and biological process.

Supplemental Figure 6

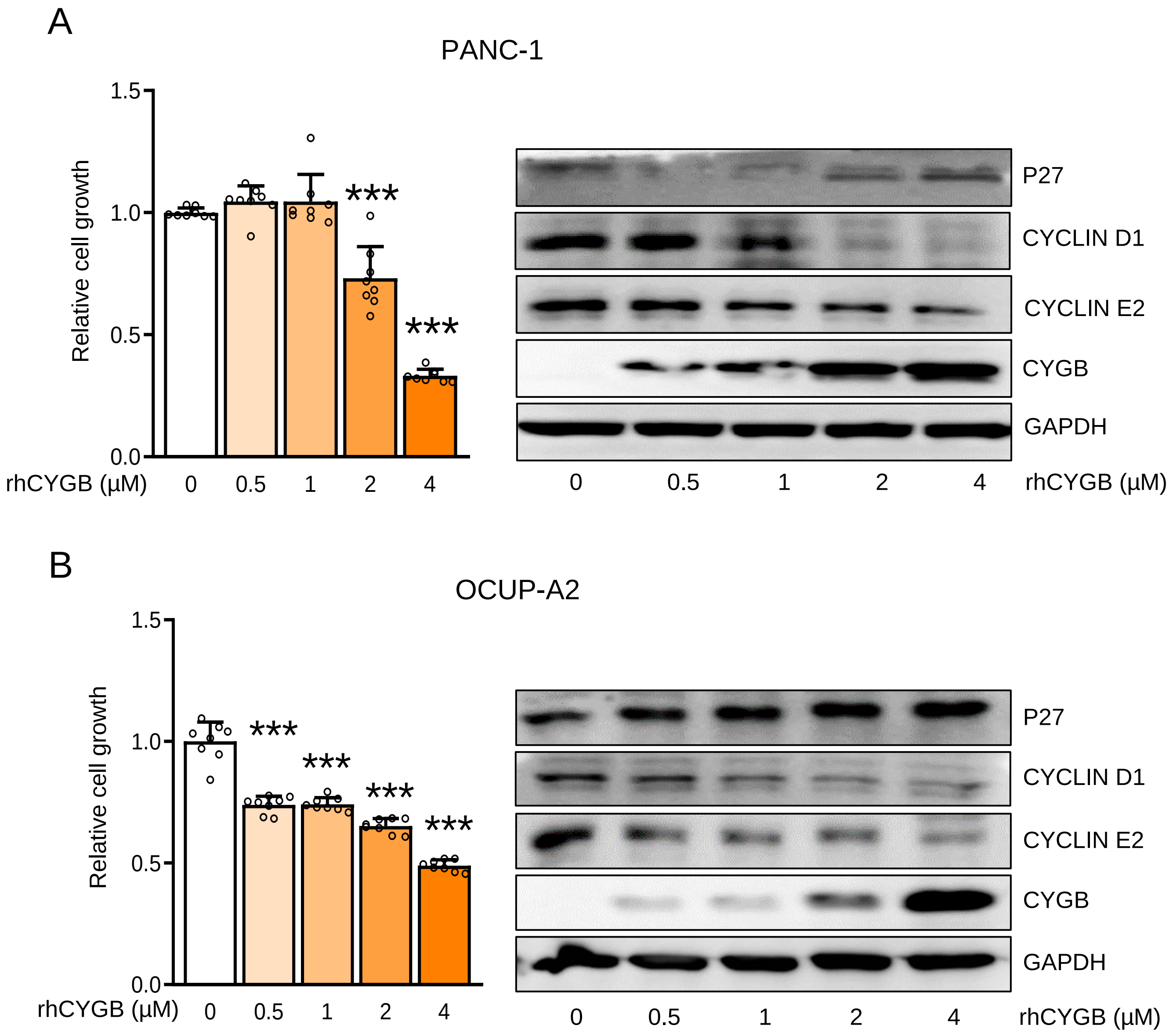

Supplemental Figure 6

rhCYGB suppressed pancreatic cancer cell growth

Determination of cell proliferation by CCK-8 assay and immunoblotting analysis of cell cycle related proteins in PANC-1 (A) or OCUP-A2 (B) treated with rhCYGB in a dose-dependent manner for 48 hours. GAPDH was used as loading control. Data were shown as mean ± SD from three independent experiments, n=8 each group. ***p < 0.001, Student's t-test.

Supplemental Figure 7

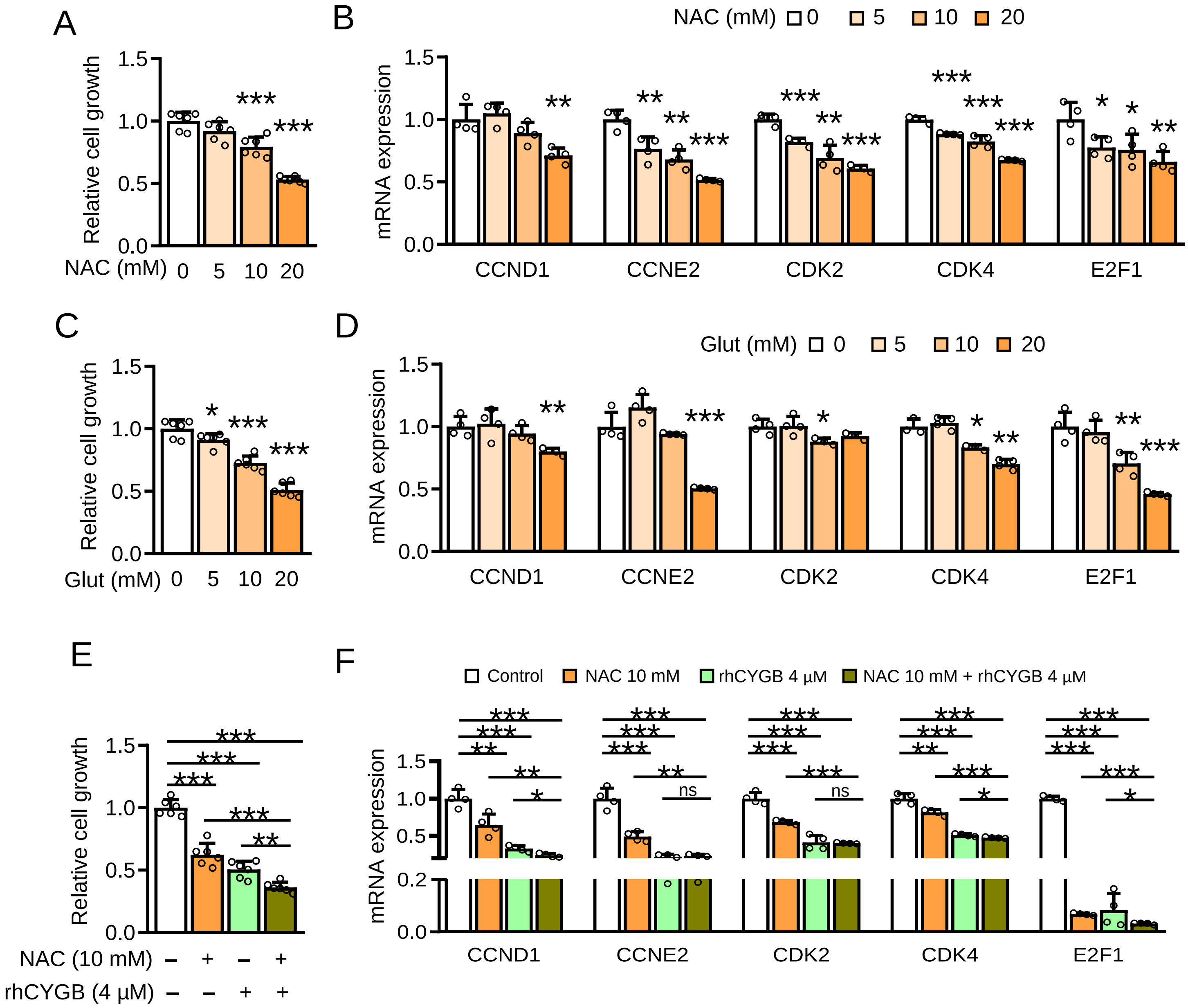

Supplemental Figure 7

Effects of N-Acetyl-L-Cysteine and Glutathione treatment separately or in combination with rhCYGB on MIA PaCa-2 cells

Determination of cell proliferation by CCK-8 assay and cell cycle related genes by qRT-PCR analysis in MIA PaCa-2 treated with N-Acetyl-L-Cysteine (NAC) (A-B) and Glutathione (Glut) (C-D) in a dose-dependent manner for 48 hours. Determination of cell proliferation by CCK-8 assay and cell cycle related genes by qRT-PCR analysis in MIA PaCa-2 treated with rhCYGB for 48 hours and followed by NAC for the last 24 hours. Data were shown as mean ± SD, n=6 each group. *p < 0.05, **p < 0.01, ***p < 0.001, Student's t-test.

Supplemental Figure 8

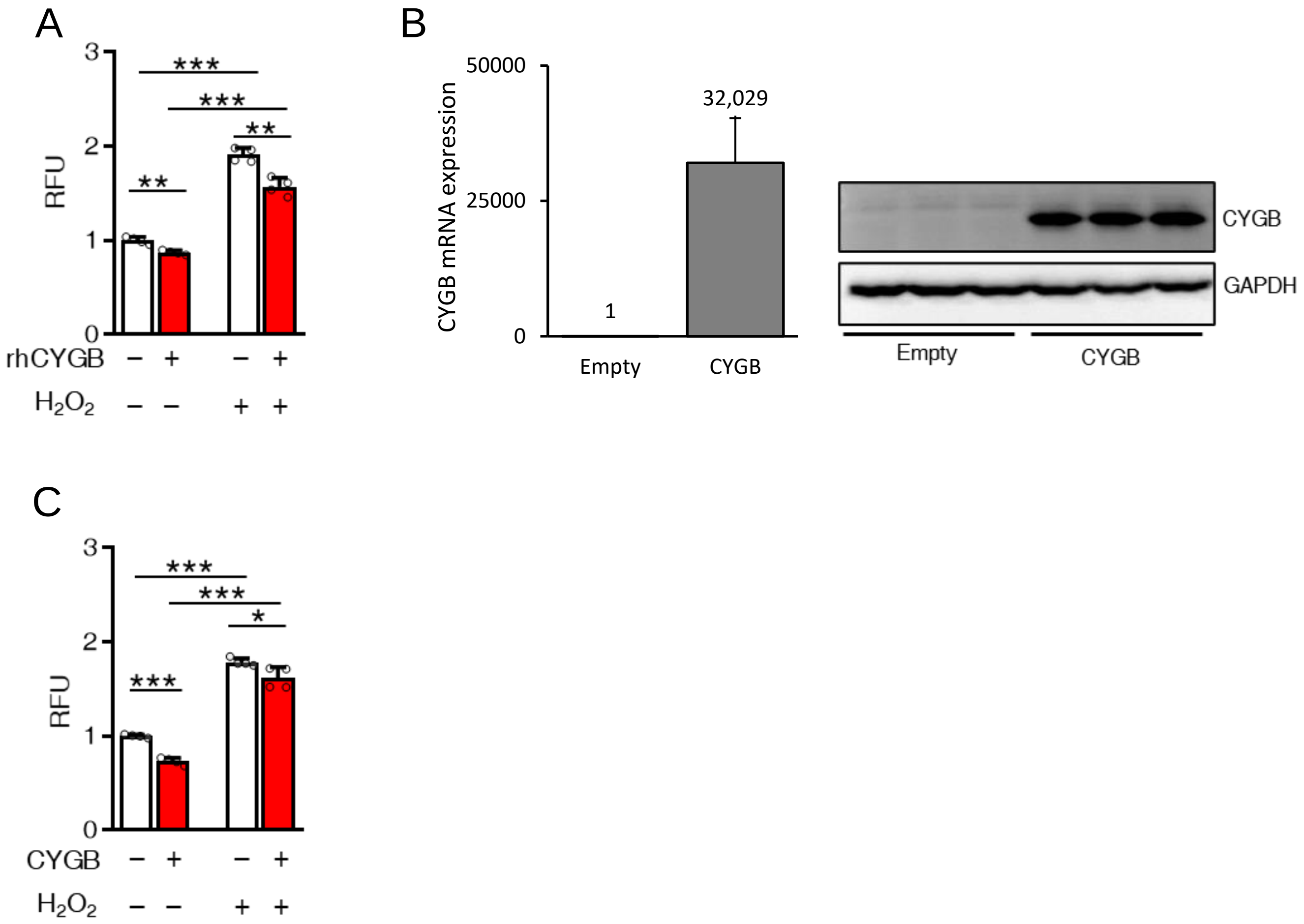

Supplemental Figure 8

ROS scavenging effect in the presence of rhCYGB or CYGB overexpression

(A) Determination of intracellular ROS by BES- H_2O_2 -Ac assay using H_2O_2 200 µM stimulation for 1 hour in MIA PaCa-2 which were 24 hours treated with or without rhCYGB 2.5 µM. (B) qRT-PCR and immunoblotting analysis of CYGB expression in MIA PaCa-2 which were 24 hours transiently transfected with empty vector (CYGB –) or pcDNA-6His-FLAG-CYGB vector (CYGB +) vector. (C) Determination of intracellular ROS by BES- H_2O_2 -Ac assay using H_2O_2 200 µM stimulation for 1 hour in MIA PaCa-2 which were 24 hours transiently transfected with empty vector or pcDNA-6His-FLAG-CYGB. Data were shown as mean ± SD from three independent experiments n=4 each group. *p < 0.05, **p < 0.01, ***p < 0.001, Student's t-test.

Supplemental Figure 9

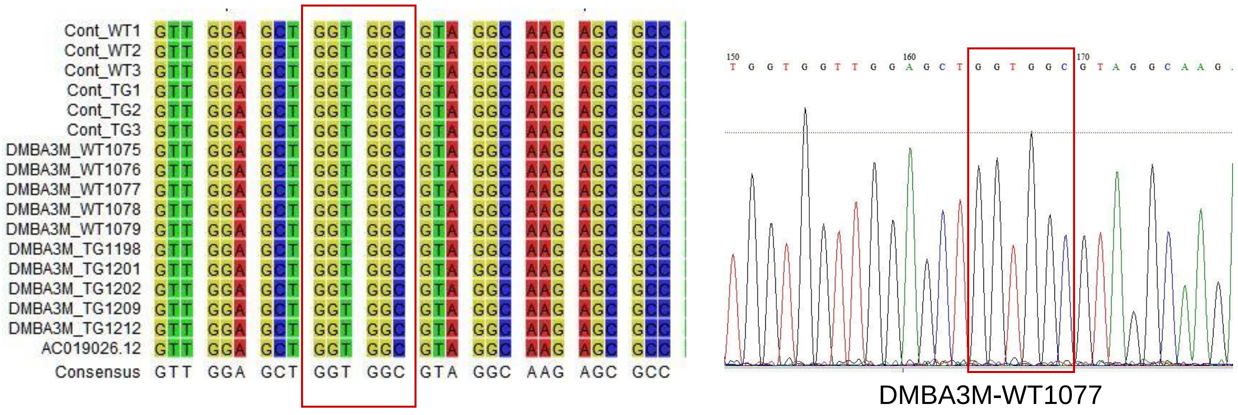

Supplemental Figure 9

No Kras mutation in control and DMBA-3M treated pancreatic tissues

Kras mutation analysis by direct sequencing of codon 12, 13 (red box) in control and DMBA-3M treated mice. AC019026.12, mus musculus chromosome 6 clone RP23-188E5 strain C57BL6/J used as reference sequence.

Supplemental Figure 10

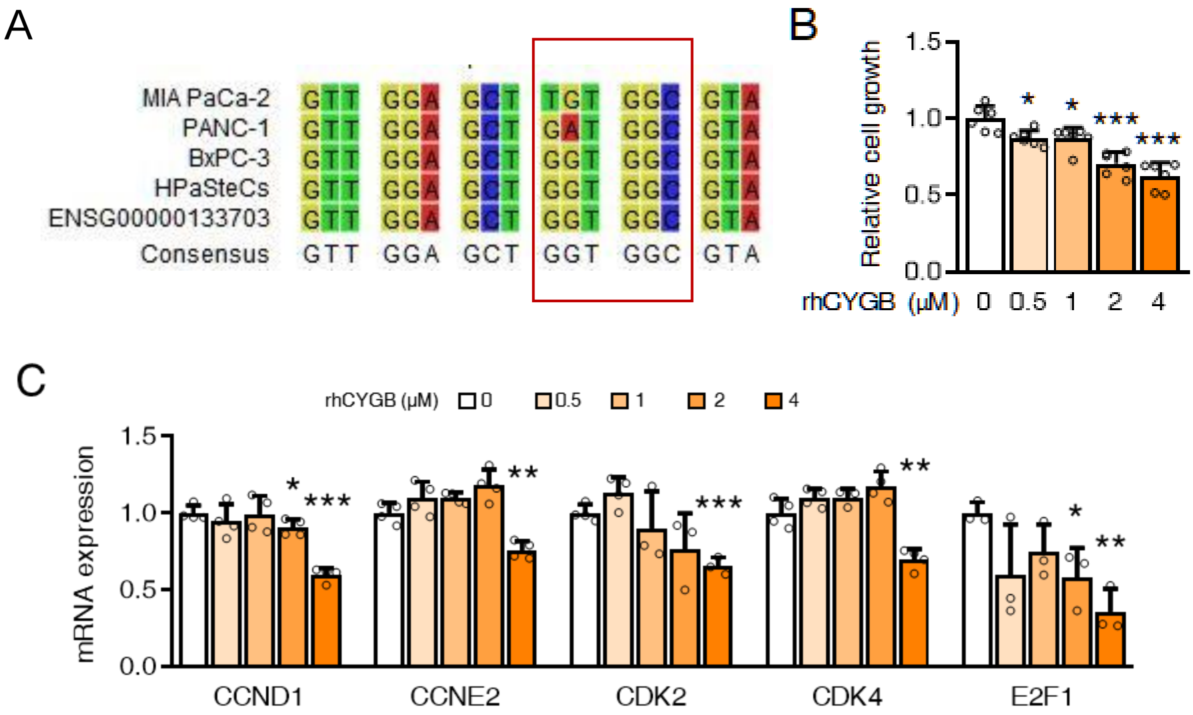

Supplemental Figure 10

KRAS mutation in pancreatic cancer cell lines and HPaSCs

(A) *KRAS* mutation analysis by direct sequencing of codon 12, 13 (red box) in pancreatic cancer cell lines, and HPaSCs. ENSG00000133703, Homo sapiens chromosome 12 used as reference sequence. Note that *KRAS* mutation at codon 12 were found in Mia PaCa-2 (from GGT to TGT) and PANC-1 (from GGT to GAT) but not found in BxPC-3 cancer cells or HPaSCs. (B-C) Determination of cell proliferation by CCK-8 assay and cell cycle related genes by qRT-PCR analysis in BxPC-3 treated with rhCYGB in a dose-dependent manner for 48 hours. Data were shown as mean ± SD, n=4 each group. *$p < 0.05$, **$p < 0.01$, ***$p < 0.001$, Student's t-test.
